# Enhanced Expression of p53 and Suppression of PI3K/Akt/mTOR by Three Red Sea Algal Extracts: Insights on Their Composition by LC-MS-Based Metabolic Profiling and Molecular Networking

**DOI:** 10.3390/md21070404

**Published:** 2023-07-17

**Authors:** Nouran M. Fahmy, Mariam I. Gamal El-Din, Maha M. Salem, Sarah H. Rashedy, Gyu Sung Lee, Yoon Seo Jang, Ki Hyun Kim, Chung Sub Kim, Mohamed El-Shazly, Shaimaa Fayez

**Affiliations:** 1 Department of Pharmacognosy, Faculty of Pharmacy, Ain-Shams University, Cairo 11566, Egypt; nouran_fahmy@pharma.asu.edu.eg (N.M.F.); mariam_gamal@pharma.asu.edu.eg (M.I.G.E.-D.); shaimaa_fayez@pharma.asu.edu.eg (S.F.); 2 Biochemistry Division, Chemistry Department, Faculty of Science, Tanta University, Tanta 31527, Egypt; maha_salem@science.tanta.edu.eg; 3 National Institute of Oceanography and Fisheries (NIOF), Cairo 11516, Egypt; sarahamdy.niof@gmail.com; 4 Department of Biopharmaceutical Convergence, Sungkyunkwan University, Suwon 16419, Republic of Korea; dlrbtjd36@skku.edu; 5 School of Pharmacy, Sungkyunkwan University, Suwon 16419, Republic of Korea; bbj0423@g.skku.edu (Y.S.J.); khkim83@skku.edu (K.H.K.)

**Keywords:** brown algae, HR-LC/MS/MS, apoptosis, PI3K/Akt/mTOR, protein kinases, cytotoxicity

## Abstract

Brown algae comprise up to 2000 species with wide dissemination in temperate zones. A comprehensive untargeted metabolic profiling guided by molecular networking of three uninvestigated Red-Sea-derived brown algae, namely *Sirophysalis trinodis*, *Polycladia myrica*, and *Turbinaria triquetra*, led to the identification of over 115 metabolites categorized as glycerolipids, fatty acids, sterol lipids, sphingolipids, and phospholipids. The three algae exhibited low-to-moderate antioxidant capacity using DPPH and ABTS assays. Preliminary in vitro antiproliferative studies showed that the algal extracts displayed high cytotoxic activity against a panel of cancer cell lines. The most potent activity was recorded against MCF-7 with IC_50_ values of 51.37 ± 1.19, 63.44 ± 1.13, and 59.70 ± 1.22 µg/mL for *S. trinodis*, *P. myrica*, and *T. triquetra*, respectively. The cytotoxicity of the algae was selective to MCF-7 without showing notable effects on the proliferation of normal human WISH cells. Morphological studies revealed that the algae caused cell shrinkage, increased cellular debris, triggered detachment, cell rounding, and cytoplasmic condensation in MCF-7 cancer cells. Mechanistic investigations using flow cytometry, qPCR, and Western blot showed that the algae induced apoptosis, initiated cell cycle arrest in the sub-G_0_/G_1_ phase, and inhibited the proliferation of cancer cells via increasing mRNA and protein expression of p53, while reducing the expression of PI3K, Akt, and mTOR.

## 1. Introduction

Nature has long been established as the cornerstone of traditional medicine, which continuously provides humanity with a plethora of metabolites with therapeutic potential against a wide spectrum of diseases [1,2]. Owing to the overconsumption of terrestrial plants, their relative depletion, and the threat of extinction of multiple species, elevated attention has recently been directed to marine natural products as a unique prolific mine of potential drug leads [3]. Constituting more than 70% of the earth’s surface, oceans and seas produce a great diversity of more than 300,000 living species, with chlorophyll-containing algae constituting more than 20,000 species [4].

Marine algae, as a copious source of minerals, vitamins, fibers, amino acids, polysaccharides, and polyunsaturated fatty acids, are globally consumed for their nutritional importance as food supplements and as sources of high-valued oils [5]. Their environmental applications are diverse and include heavy metal sequestering, water treatment, coloring agents, food additives, and preparation of hydrocolloids [6]. Miscellaneous secondary metabolites were isolated from algae, which exhibited propitious bioactivities including antimicrobial, anti-inflammatory, cytotoxic, antioxidant, anticoagulant, and antifungal effects [7,8,9]. Several algal-driven constituents were patented and marketed for nutritional and medicinal uses [10]. The diversity and pharmacological benefits of marine algae were our motive for in-depth mining for algal species, especially from the Red Sea, with its unique geography between Africa and Asia. The Red Sea is regarded as a valuable reservoir for a wide and unexplored marine ecosystem [11]. 

Brown algae (Phaeophyta) are characterized by the domination of fucoxanthin, a brown xanthophyll pigment imparting them with their color, and constitute a significant proportion of the Red Sea marine ecosystem [12]. Brown algae comprise a complex mixture of marine organisms extending from microscopic filamentous thallus to complex macroalgae. Phlorotannins, fucoxanthins, lanimarins, alginates, fatty acids, prostaglandins, sterols, and fucoidans are among the major secondary metabolites characterized by the different species of brown algae and they are probably responsible for their reported biological activities [13]. Despite comprising more than 2000 species [11], only a few of these brown algae were extensively studied for their chemical and biological properties.

Cancer poses a significant socioeconomic burden and a critical public health issue, especially in developing nations [14]. It is currently considered as the 3rd leading cause of mortality with more than 17 million cancer-related deaths, and over 26 million new cases are estimated to emerge by 2030 [15]. Conventional measures of treatment such as surgery, radiation, and chemotherapy are usually associated with dramatic side effects, raising an urge for alternative medicines with better safety profiles [16]. The PI3K/Akt/mTOR signaling axis, defined by phosphatidylinositol-3 kinase (PI3K), protein kinase B (Akt), and mechanistic target of rapamycin (mTOR), is a complex pathway with several upstream regulators and downstream effectors that play a key role in cancer pathology [17]. The PI3K/Akt/mTOR signaling axis is involved in a wide variety of cellular biological activities including cell growth, metastasis, survival, and metabolism. Inhibitors targeting important kinases of the PI3K/Akt/mTOR pathway have received increased interest from the scientific community [18].

The three brown algae, *Turbinaria triquetra*, *Sirophysalis trinodis*, and *Polycladia myrica*, are abundant species in the Red Sea and they belong to the Family Sargassaceae. *P. myrica*, also spreads along the Mediterranean Sea, the Pacific, and Indian Oceans [19]. Previous studies reported in vivo antidiabetic activities of the crude methanol extracts of *S. trinodis* and *P. myrica*, where the α-glucosidase inhibition IC_50_ values were 0.33, 3.50, and 3.31 μg/mL for *T. triquetra*, *S. trinodis*, and *P. myrica*, respectively, compared with 160.15 μg/mL for the standard acarbose. Additionally, *S. trinodis* reduced the postprandial blood glucose levels in diabetic rats [20]. Antioxidant and antibacterial effects were reported for the extracts of *T. triquetra* and *P. myrica*. The methanol extract of *T. Triquetra* and the petroleum ether extract of *P. myrica* were more active against Gram-negative bacteria with MIC values ranging from 0.5 to 2 µg/mL [19,21,22]. *P. myrica* displayed protective effects against UV-radiation, where a 5% formulated cream of *P. myrica* displayed a protective factor of 31.79  ±  4.73 [23]; however, nothing was detected in the literature concerning the cytotoxic potential of the selected brown algal species. Therefore, in the current study, the chemical metabolome of the crude extracts of *T. triquetra*, *S. trinodis*, and *P. myrica* was traced using untargeted HR-MS/MS profiling guided by GNPS molecular networking. Their antioxidant activities were studied using DPPH and ABTS assays. Preliminary cytotoxic studies were performed on several cancer cell lines, supported by extensive mechanistic investigations of their anti-proliferative effect using flow cytometry, qPCR, and Western blot analysis.

## 2. Results and Discussion

### 2.1. Untargeted Metabolome Analysis

**Figure 1:**
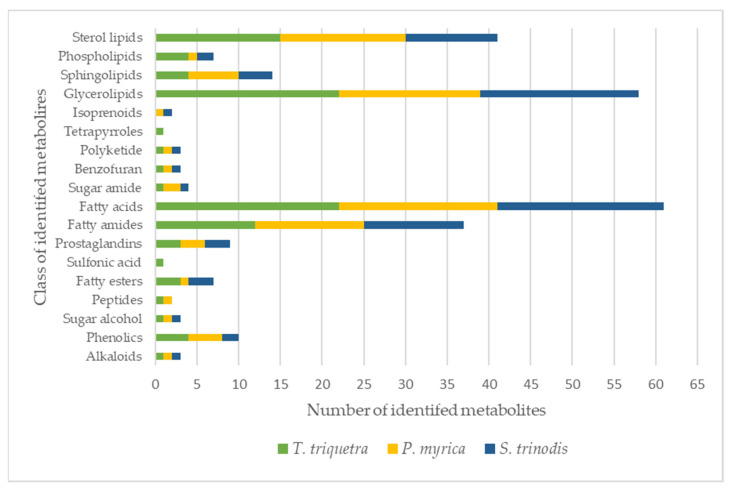
Classes of metabolites identified in the three Red Sea algal species. The colors in each bar represent the total number of metabolites per algae.

Detailed metabolomic analysis of the crude extracts of the three Red Sea algal samples measured in the positive and negative ion modes (for chromatograms see Figures S7–S9) resulted in the annotation of over 115 metabolites (with mass errors less than 5 ppm), including 25 fatty acids and their hydroxylated derivatives, 27 glycerolipids, 17 sterol lipids, 15 fatty amides, 6 sphingolipids, and 4 phospholipids (Table 1 and Figure 1). The current study is the first report elucidating the chemical metabolic profile of three Red-Sea-derived brown algae viz, Turbinaria triquetra, Sirophysalis trinodis, and Polycladia myrica using untargeted liquid chromatography-high-resolution mass spectrometry (LC-HRMS).

#### 2.1.1. Glyceroglycolipids

Glyceroglycolipids represented the major class of metabolites identified in the studied algae species with a total number of 27 compounds identified in the three species. Glyceroglycolipids, with their glycerol backbone attached to one or more glycosyl moieties and different acyl groups, are known to be highly abundant in marine algae and are responsible for a wide array of biological activities [24,25]. Monoacyl glycerol was detected either free or in conjugation with monogalactose (MGMG at t_R_ 14.3, 14.5, 16.0, 16.5, and 21.0 min), digalactose (DGMG at t_R_ 12.4 and 15.0 min), trimethyl homoserine (at t_R_ 13.1, 14.3, and 14.8 min), glucuronic acid (t_R_ at 15.9, 17.8, 18.4, 21.4, and 22.9 min), palmitoyl galactosyl, or sulfoquinovosyl moiety (SQMG at t_R_ 14.06, 14.3, 15.04, 16.9, and 17.3 min). On the other hand, free diacylglycerol was not freely detected but only conjugated with glucuronic acid (DGGA at t_R_ 15.3 min), monogalactose, or carboxyhydroxymethylcholine moieties. The relative distribution of the different glycerolipids [(i.e., monogalactosyldiacylglycerol (MGDG), digalactosyl-diacylglycerol (DGDG), and sulfoquinovosyl-diacylglycerol (SQDG)] were close to that observed in a previous study performed on a collection of brown algae from the Black Sea with ranges of MGDG, DGDG, and SQDG estimated as 26.8–46.5%, 19.6–44.1%, and 17.5–51.7%, respectively [26]. It is worth mentioning that the highest number of glycerolipids was detected in *T. triquetra* with a total of 22 vs. 19 and 17 in *S. trinodis* and *P. myrica*, respectively.

#### 2.1.2. Fatty Acids and Hydroxy Fatty Acids

**Figure 2:**
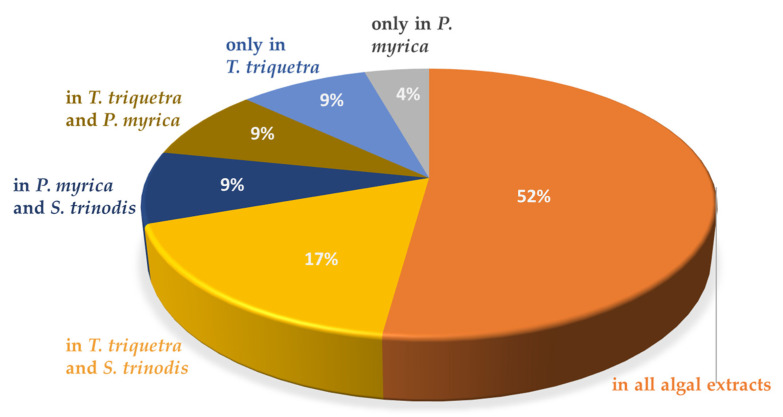
Distribution of fatty acids in the Red Sea algal extracts.

HR-LCMS analysis revealed the predominance of fatty acids and their hydroxides in the three algal extracts with chain lengths ranging between C_9_ and C_29_. A total of 25 fatty acids were detected, constituting ca. 21% of the identified chemical constituents. The highest number of fatty acids are present in *T. triquetra* with a total of 22 vs. 20 for *S. trinodis* and 19 for *P. myrica*. Unsaturated fatty acids were more prominent in the three algae, whereas hydroxyeicosanoic acid was the only saturated fatty acid identified in *T. triquetra* and *P. myrica*. Twelve fatty acids were common in the three algae (but with different concentrations; see Table 1) including trihydroxyoctadecadienoic acid (TriHODE), trihydroxyoctadecenoic acid (TriHOME), hydroxyoctadecenoic acid, hydroxyoctadecadienoic acid (HODE), hydroxyoctadecatrienoic acid, dihydroxyhexadecenoic acid, undecanedioic acid, undecatrienoic acid, eicosapentaenoic acid, hydroxyeicosapentaenoic acid, hydroxyeicosatetraenoic acid, hydroxyeicosadienoic acid, and dihydroxyeicosatetraenoic acid, representing ca. 23% of the total identified fatty acids. Azelaic acid, octadecatrienoic acid, hydroxynonacosahexaenoic acid, and hydroxyoctadecatetraenoic acid were only observed in *T. triquetra* and *S. trinodis*, while dodecanedioic acid and hydroxydioxoheptadecenoic acid were solely detected in *P. myrica* and *S. trinodis*. Octadecenoic acid and hydroxyeicosanoic acid were only identified in *T. triquetra* and *P. myrica*. It is worth mentioning that the two fatty acids dihydroxyoctadecatrienoic acid and hydroxynonacosaheptaenoic acid could be used as markers for discriminating *T. triquetra* from the other two species. Dihydroxyoctadecenoic acid was solely detected in *P. myrica*. Figure 2 illustrates the distribution of identified fatty acids as a percentage in each algal extract.

Unsaturated fatty acids were more prominent in the three algae extracts, with hydroxyeicosanoic acid representing the only saturated fatty acid identified in both *T. triquetra* and *P. myrica* extracts. The results were in agreement with a previous study reporting the richness of *S. trinodis* collected from the Persian Gulf, with polyunsaturated fatty acids being primarily oleic acid and arachidonic acid [27]. Another study performed on *P. indica* and *T. ornata* macroalgae collected from the Red Sea shore in Egypt reported that oleic acid was the major unsaturated fatty acid in both brown seaweeds [28]. On the other hand, a study on *S. trinodis* and *P. myrica* from the Persian Gulf reported the presence of saturated fatty acids including myristic, palmitic, stearic, and arachidic acids, where the total saturated fatty acids constituted 41% and 45% of the estimated fatty acid composition in *S. trinodis* and *P. myrica*, respectively [29].

#### 2.1.3. Sterol Lipids

Sterol lipids constituted the third major class of the identified constituents after glycerolipids and fatty acids with a total of 17 identified compounds. Sterols could be free or conjugated with glycine, hexose, or sulfate. Most identified sterols were free, having different degrees of saturation and oxygenation. Four of the unconjugated sterols were saturated (at t_R_ 15.7, 15.8, 19.6, and 21.9 min) and present in the three algae. The remaining five sterols were unsaturated (at t_R_ 18.2, 19.0, 20.2, 22.6, and 22.8 min), three of which were observed only in *T. triquetra* and *P. myrica*. Two sterols were conjugated with glycine (t_R_ 6.8 and 17.8 min), one was conjugated with sulfate (t_R_ 14.5 min), and two were conjugated with hexose (t_R_ 15.5 and 20.2 min). There were three other sterols (t_R_ 18.4, 18.8, and 19.7 min) that might be either hexose-conjugated or free, and were detected in the three algae. Glycine and hexose-conjugated sterols were at the highest amounts in *P. myrica*, whereas the only sulfate-conjugated sterol was found in *T. triquetra*. Overall, *T. triquetra* and *P. myrica* showed an almost similar content of sterol lipids, while *S. trinodis* demonstrated the lowest number of sterol lipids. Sterol lipids were previously identified in many brown algae species [30,31]. Previous studies reported sterols as a major prevailing class of marine metabolites responsible for different biological activities, particularly in the genus *Turbinaria* [32,33,34].

#### 2.1.4. Fatty Amides

A total of 15 fatty amides were detected, 9 of which were found in the three algal samples such as oxododecanoyl homoserine (t_R_ 5.9 min), stearoyl phenylalanine (t_R_ 11.8 min), palmitoyl tryptophan (t_R_ 15.1 min), linoleamide (t_R_ 17.1 min), oleamide (t_R_ 19.1 min), oleoyl alanine (or might be palmitoyl proline, t_R_ 19.5 min), oleoyl GABA (t_R_ 20.3 min), octadecanamide (t_R_ 21.3 min), and palmitoyl valine (t_R_ 21.6 min). Acyl- (t_R_ 16.5 min) and lauroyl- (t_R_ 17.7 min) carnitine were only observed in *P. myrica*, while palmitoyl alanine (t_R_ 19.0 min) and palmitoyl (iso)leucine (t_R_ 22.2 min) were solely detected in *T. triquetra* and *S. trinodis*. N-acyl glycine 28:6 (t_R_ 13.2 min) was only identified in *S. trinodis*. The three algae had an almost similar percentage of fatty amides. Oleamide has been previously detected in the alcohol-macerated extract of *Turbinaria ornata* when analyzed by LC-HRMS, along with other amides and fatty acid esters including myristamide, erucamide, arachidonic acid ethyl ester, and γ-linolenic acid ethyl ester [35].

#### 2.1.5. Sphingolipids

Sphingadienine (t_R_ 14.6 min), sphingatrienine (t_R_ 13.6 min), N-tridecanoyl-tetradecasphingenine (t_R_ 15.2 min), deoxysphingatetraenine (t_R_ 15.3 min), N-pentadecenoyl-tetradecasphingenine (t_R_ 17.2 min), and hexose ceramide 37:4;O4 (t_R_ 18.4 min) were the six sphingolipids detected in the algal samples. Sphingatrienine and hexose ceramide were solely identified in the extract of *P. myrica*, whereas the remaining sphingolipids were detected in the three algae. Our results agreed with the previous literature reporting the relatively low abundance of sphingolipids in microalgae relative to other types of lipids [36,37,38].

#### 2.1.6. Phospholipids

Phospholipids generally belong to one of the two major subclasses which are phosphatidylcholines and phosphatidylglycerols. Many of these were reported in different brown algae from the Black Sea [26]. Four phospholipids were identified in the current study representing the two abundant classes in the marine ecosystem. Phosphatidylglycerol 20:1 (t_R_ 16.0 min) was solely detected in *T. triquetra*, while its oxidized form PG 22:4;O3 (t_R_ 21.4 min) was observed in both *T. triquetra* and *P. myrica*. Phosphatidylcholines such as lysophosphatidylcholine and its ether form were only observed in *T. triquetra* and *S. trinodis.* Phosphatidylglycerol and lysophosphatidylcholine were previously identified from different brown algae, viz. *Undaria pinnatifida* and *Laminaria japonica* [39,40].

#### 2.1.7. Fatty Esters and Fatty Alcohols

Three fatty acid esters and one fatty alcohol were identified. A fatty acid ester of a hydroxy fatty acid (t_R_ 18.19 min) and the phenolic fatty alcohol dodecylphenol (t_R_ 16.99 min) were detected in all three algal extracts, however, stearyl citrate (t_R_ 16.58 min) and palmitoyl hexitol (t_R_ 18.17 min) were identified only in *T. triquetra* and *S. trinodis*. Saturated fatty alcohols had previously been reported from *Turbinaria reniformis* [31].

#### 2.1.8. Phenolics

Six different phenolic compounds, including flavonoid, benzofuran, sulfonic acid derivative, and simple phenols were identified in the examined algae. Formononetin (t_R_ 2.02 min), an isoflavone, was detected in *T. triquetra* and *P. myrica* extracts. The benzofuran analog (iso)loliolide (t_R_ 7.07 min) was observed in all algae; however, the sulfonic acid derivative, N-undecyl benzene sulfonic acid (t_R_ 20.91 min) was identified only in *T. triquetra*. Simple phenolics such as dimethoxyphenyl propene (t_R_ 7.07 min), 4-dodecyl phenol (t_R_ 16.99 min), and trimethylphenyl butanone (t_R_ 8.37 min) were detected, with the former two compounds being observed in all algae and the latter one being identified in *T. triquetra* and *P. myrica*. Our results agreed with a previous study estimating the total phenolic content of the methanol extract of *T. triquetra* as 1218.51 ± 27.5 µg gallic acid equivalent /mL and the study reported the relative abundance of phenolic constituents in brown macroalgae compared with the green and red seaweeds [33,41].

#### 2.1.9. Prostaglandins

Three prostaglandins were identified in the algae samples. Prostaglandin E_2_ (t_R_ 9.30 min) and F_2_ (t_R_ 10.0 min), and hydroxy PGE_1_ (or possibly hydroxy PGF_2α_, t_R_ 9.0 min) were detected in all three Red Sea algal extracts. PGE_2_ was previously identified from the brown algae Laminaria digitata and was suggested to play a defensive role against copper-induced oxidative stress [42].

#### 2.1.10. Short-Chain Peptides

Two short-chain peptides were recognized: the first one was composed of glutamic acid, histidine, and threonine (t_R_ 2.21 min) and was only detected in *T. triquetra*, whereas the second peptide (t_R_ 15.75 min) was identified solely in *P. myrica* and was composed of arginine and two lysine amino acid units. The glycine amino acid was observed as a conjugate with several sterols having different unsaturation and oxygenation patterns, as previously discussed.

#### 2.1.11. Alkaloids

The anthranilic-acid-derived alkaloid, quinoline carboxylic acid (t_R_ 1.93 min), was only detected in *S. trinodis*, while the quinolizidine alkaloid acrifoline (t_R_ 11.81 min) was only observed in *T. triquetra*. On the other hand, the tetrahydro *β*-carboline alkaloid antirhine (t_R_ 12.23 min) was detected in *P. myrica*.

#### 2.1.12. Sugar Alcohols and Sugar Amides

The sugar alcohol galactitol (t_R_ 2.08 min) and the sugar amide carbamoyl aminodeoxyhexitol (t_R_ 10.07 min) were detected in all algae; however, glucaramide (t_R_ 9.13 min) was solely detected in *P. myrica* extract.

#### 2.1.13. Terpenes (Isoprenoids)

The plant hormone abscisic acid (t_R_ 12.60 min) and the hydroazulene diterpene dictyone acetate (t_R_ 16.18 min) were the only terpenes detected in the algal extracts.

#### 2.1.14. Miscellaneous Compounds

The polyketide macrolide deoxyerythronolide B (t_R_ 19.41 min), was detected in all three algae, while the chlorophyll derivative phaeophorbide “a” (t_R_ 20.11 min) was only observed in *T. triquetra*.

### 2.2. HR-MS/MS-Based Molecular Networking

**Figure 3:**
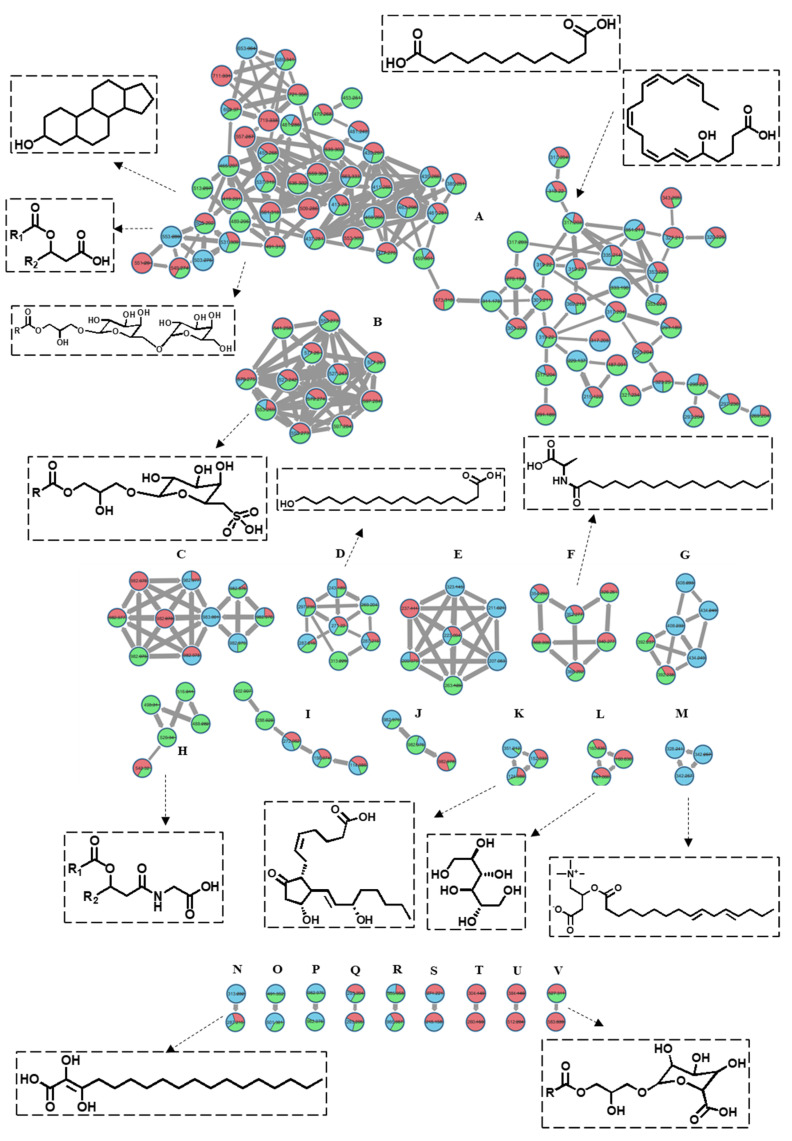
The molecular network of the annotated metabolites in three Red Sea algal samples (established in the negative mode) shows 261 nodes and 22 clusters. The nodes are labeled with their parent masses. Those nodes showing similar parent masses indicate isomerism. The network is displayed as a pie chart to reflect the relative abundance of each ion in the analyzed samples. *Turbinaria triquetra* is indicated in red, *Polycladia myrica* in blue and *Sirophysalis trinodis* in green.

The tandem spectroscopic mining based on LC-MS^2^ data associated with the online GNPS (Global Natural Products Social Molecular Network) platform was carried out to build a molecular network that clusters molecules with matched MS/MS fragments, allowing the direct visualization of the metabolic profile of the three studied algae. The network, which is established based on the negative ionization data, consisted of 261 nodes comprising 22 clusters and 48 self-looped nodes (Figure 3). The dereplication of the nodes was performed manually and using the GNPS library. Each node, labeled with the molecular ion peak, was plotted as a pie chart showing its relative abundance in each alga. The nodes were color-coded, where blue stands for *P. myrica*, red for *T. triquetra*, and green for *S. trinodis*. Cluster A was the largest in the molecular network and constituted mainly of lipids with a closely related fragmentation pattern. The nodes in cluster A were classified into two major groups: The first one elucidated the presence of nine sterol lipids, three fatty esters, and nine glycerolipids primarily belonging to DGMG, MGGA, or MGMG. The second group of nodes exemplified the predominance of fatty acids, where 22 of them were outlined based on matched fragments. 

Cluster B comprised eight nodes, six of which were identified as SQMG, indicating the presence of glycerolipids conjugated with a sulfoquinovosyl moiety. The presence of characteristic fragments at *m*/*z* 80.965 and/or *m*/*z* 225.006 confirmed their identity. These compounds were concentrated in *T. triquetra* and *S. trinodis* when compared with *P. myrica*. Cluster D showed seven nodes, six of which were identified as fatty acids. Cluster F grouped six nodes, five of which showed parent masses at *m*/*z* 352.27, 326.26, 354.29, 368.31, and 366.292, and were identified as fatty amides, as evidenced by the presence of common fragments at *m*/*z* 116.07 and 130.08. Cluster H displayed sterol glycine lipid conjugated with a characteristic fragment ion at *m*/*z* 78.95.

Cluster K showed a precursor ion peak at *m*/*z* 351.212, annotated as prostaglandin E_2_, while cluster L reflected the presence of a sugar alcohol at *m*/*z* 181.065, identified as galactitol. These compounds were traced only in *T. triquetra* and *S. trinodis*. Cluster M proposed the presence of acylcarnitine derivatives which were exclusively detected in *P. myrica*. Closely related hydroxy fatty acids were clustered together. One example is dihydroxyoctadecenoic acid and dihydroxyhexadecanoic acid, which form cluster N and have a mass difference of 26 Da. Cluster U comprised two nodes that are exclusively present in *T. triquetra*, one of which belonged to peptides and was identified as Glu-His-Thr. Cluster V showed the presence of monoacylglycerylglucuronides (MGGA).

### 2.3. Assessment of the Antioxidant Activity Using DPPH and ABTS Assays

**Figure 4:**
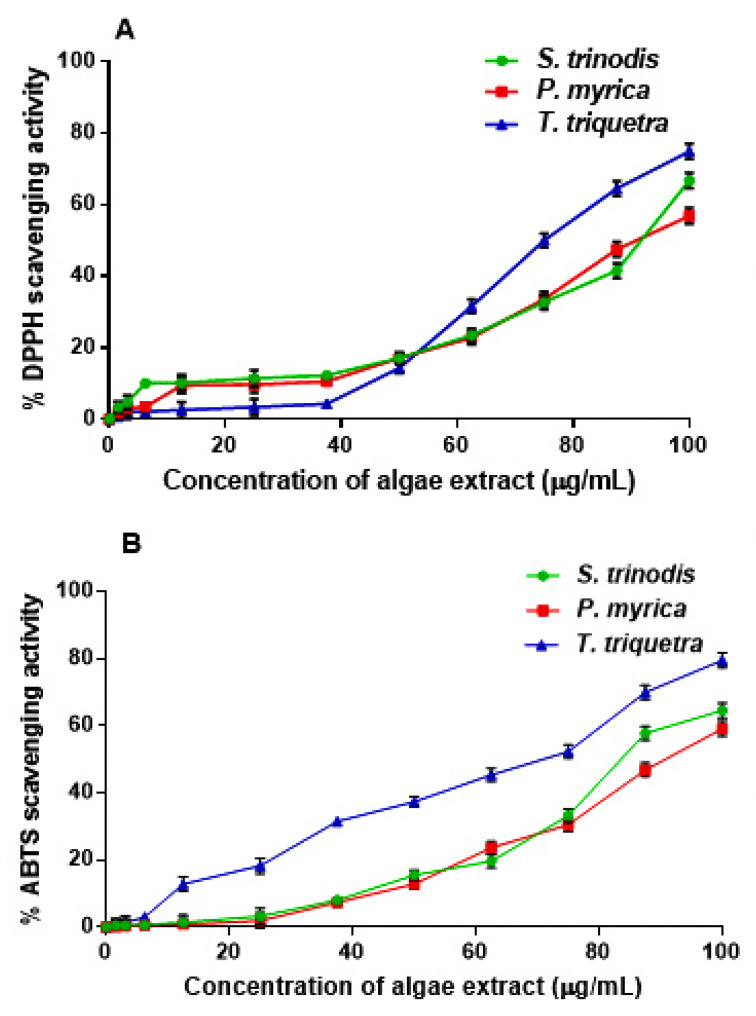
Assessment of the free radical scavenging activity of the crude extracts of *S. trinodis*, *P. myrica*, and *T. triquetra* using DPPH (**A**) and ABTS (**B**) assays. Results were expressed as mean ± SE, (n = 3).

The free radical scavenging potential of the crude methanol extracts of the three Red Sea algae were assessed in vitro using the DPPH and ABTS assays (Figure 4). The three algae, *S. trinodis*, *P. myrica*, and *T. triquetra*, displayed weak-to-moderate antioxidant activities with IC_50_ values of 90.78 ± 1.52, 93.62 ± 1.74, and 75.71 ± 1.84 µg/mL, respectively, in the DPPH assay, compared with the standard L-ascorbic acid (IC_50_ value of 20.3 ± 0.14 µg/mL). In the ABTS assay, the antioxidant effect was also concentration-dependent, with IC_50_ values of 85.18 ± 1.12, 91.34 ± 1.71, and 61.20 ± 1.12 µg/mL, respectively, in comparison with L-ascorbic acid (IC_50_ value of 15.7 ± 0.21 µg/mL). Despite their relatively weak antioxidant properties, some reports from the literature showed antioxidant and preservative properties of the water extracts of *P. myrica* and *S. trinodis* on treated corn stacks within three months of storage at ambient temperature [29]. The antioxidant and antiproliferative activities were also reported for the extracts of *Turbinaria conoides* and *Turbinaria ornata* [43].

### 2.4. In Vitro Assessment of Cytotoxicity

The cytotoxic activity of the three algal extracts was assessed against a panel of cancer cell lines including MCF-7, MDA-231, Caco-2, and PANC-1, as well as normal WISH human cells to assign their selectivity (Table 2). The breast cancer MCF-7 cells were highly sensitive to the algal extracts, which demonstrated significant selectivity to cancer cells without causing toxicity to normal WISH cells, compared with tamoxifen (TAM), which was extremely toxic even to normal cells. The extract of *S. trinodis* displayed the highest cytotoxicity against MCF-7 followed by *T. triquetra* and then *P. myrica*. Therefore, the MCF-7 cells were accordingly chosen for further mechanistic investigations.

### 2.5. Assessment of the Effect of the Algal Extracts on Cancer Cell Morphology

**Figure 5:**
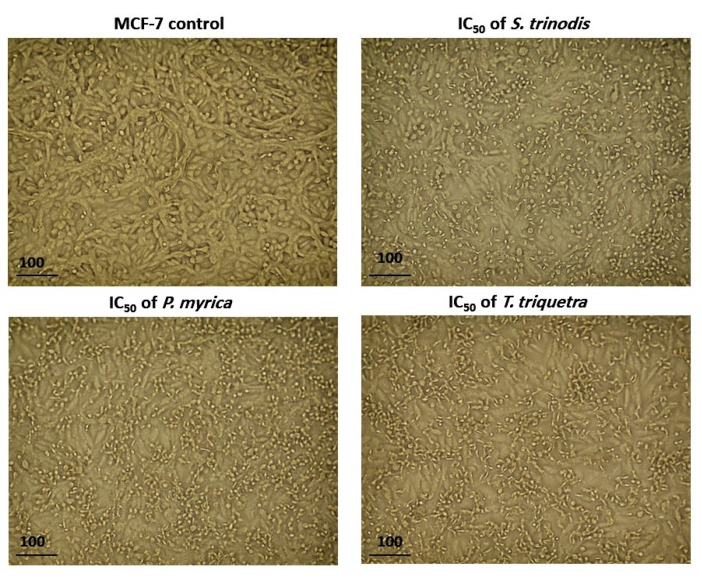
Morphological alterations in MCF-7 cells after 48 h of treatment with the three algal extracts at their IC_50_ concentrations.

After 48 h of treatment of MCF-7 cells with the algal extracts at their IC_50_ concentration, cell shrinkage, increased cellular debris, detachment, cell rounding, and cytoplasmic condensation were observed under a light-inverted microscope. The untreated cells, on the other hand, appeared normally spindle in shape and confluent (Figure 5).

### 2.6. Cell Cycle Analysis Using Flow Cytometry

**Figure 6:**
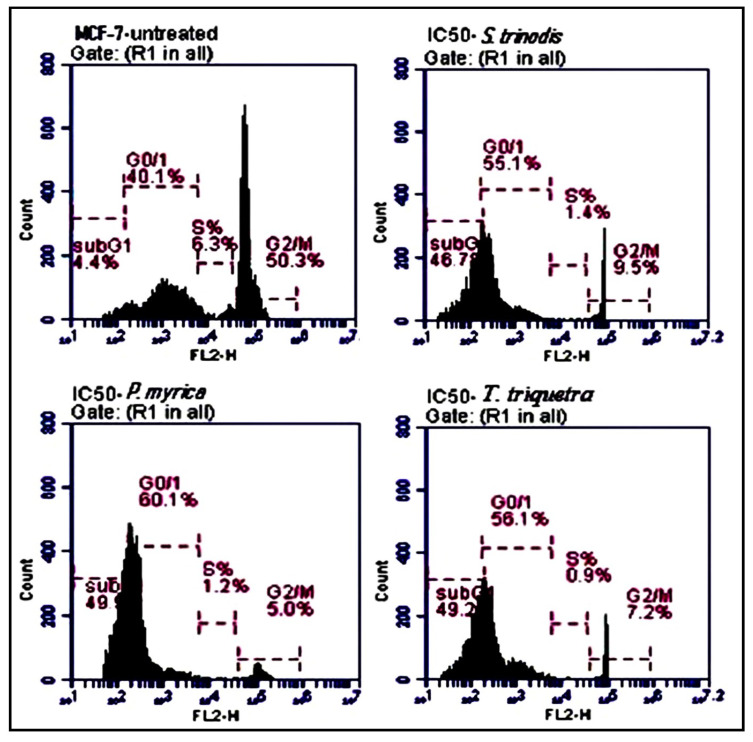
Analysis of the population of MCF-7 cells at different phases of the cell cycle before and after 48 h of treatment with the three Red Sea algal extracts at their IC_50_ concentrations. Results are expressed as mean ± SE, (n = 3).

Compared with the untreated MCF-7 cells, the algal extracts (at their IC_50_ concentrations) induced apoptosis by increased the percentage of MCF-7 cells in the sub-G_0_/G_1_ phase. The treated cells with *S. trinodis*, *P. myrica*, and *T. triquetra* caused cell cycle arrest at rates of 46.7%, 49.3%, and 49.2%, respectively (Figure 6), compared with the untreated MCF-7 cells (4.4%).

### 2.7. Real-Time Quantitative Reverse Transcription (qRT-PCR) Analysis

**Figure 7:**
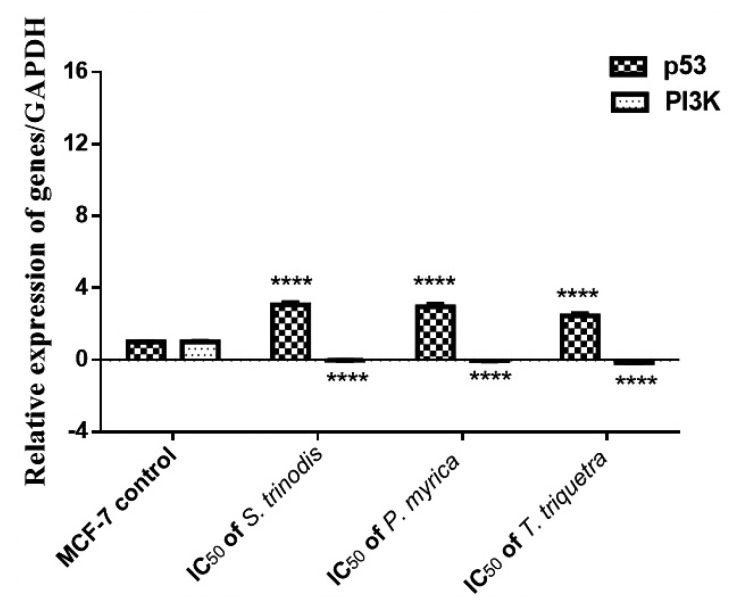
Relative expression of p53 and PI3K in MCF-7 cell lines before and after 48 h of treatment with the three Red Sea algal extracts at their IC_50_ concentrations. Results were expressed as mean ± SE, (n = 3). **** *p* < 0.0001 is considered significant compared with the MCF-7 control untreated cells.

The pivotal role of the PI3K/Akt/mTOR intracellular signaling pathway was extensively investigated in the regulation of the cell cycle and oncogenic transformation. The mRNA expression of p53 and PI3K genes in MCF-7 cancer cells was measured using quantitative real-time polymerase chain reaction (qRT-PCR). The expression of the tumor suppressor gene p53 was significantly (*p* < 0.0001) increased in cells treated with the algal extracts; however, the expression of the PI3K gene was considerably (*p* < 0.0001) downregulated in the treated cells compared with the untreated ones (Figure 7).

### 2.8. Immunoblotting Assay

**Figure 8:**
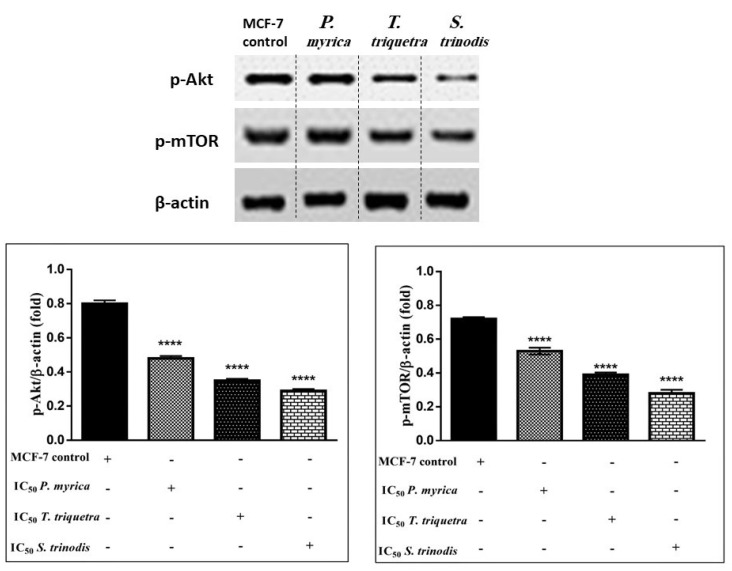
Western blotting analysis reveals the efficacy of the three algal extracts, in particular *S. trinodis*, in reducing the levels of phosphorylated Akt and mTOR proteins in MCF-7 cells. Results were expressed as mean ± SE, (n = 3). **** *p* < 0.0001 is considered significant compared with the MCF-7 control untreated cells. Bands were relatively expressed to *β*-actin protein (internal control) (Tables S1 and S2) by Western blot analysis.

As the phosphorylation of PI3K activates Akt (protein kinase B) and other downstream proteins like mTOR (mammalian target of rapamycin), the algal extracts (in particular, *S. trinodis*, which displayed the highest potency) caused significant inhibition to p-Akt (Figures S1–S3) and p-mTOR (Figures S4–S6) in MCF-7 cancer cells compared with the untreated ones. These findings revealed that algal extracts probably decreased the phosphorylation of PI3K, hence preventing Akt activation. Once Akt is dephosphorylated, it enhances the release of p53, which suppresses cancer cell proliferation and induces apoptosis (Figure 8).

The promising antiproliferative activity of the studied algal extracts against MCF-cells, manifested through the different mechanistic investigations, is most probably attributed to their rich chemical metabolic profiles. Fatty acids, a major class of the three algal extracts, were reported to enhance the death of tumor cells through mechanisms related to the changes they impart in mitochondrial transmembrane potential, reducing the production of hydrogen peroxide, and inducing mitochondrial uncoupling [44]. Glycerolipids were also reported to possess potent cytotoxic activities through induction of apoptosis and inhibition of DNA polymerase [24,45]. Moreover, several sterols isolated from different *Turbinaria* species and other brown algae demonstrated prominent antiproliferative activities against different cancer cell lines [46,47]. A tautomeric sterol isolated from *Vernonia amygdalina* Delile demonstrated significant cytotoxic activity against Hela cells via modulating the PI3K/Akt/mTOR signaling pathway. Reduced phosphorylation levels of PI3K and Akt, besides the significant reduction of phosphorylated mTOR and its substrate ribosomal kinase, were responsible for the antiproliferative activity of the evaluated sterol [48]. Cytotoxic activities were likewise reported to different minor metabolites identified in the studied alga extracts including sphingolipids, phospholipids, and phenolics [49,50,51]. The antiproliferative potential of phenolic compounds was attributed to their direct influence on cell cycle arrest at different phases and mechanisms affecting the G1, G2, and M phases [52,53]. The antioxidant activity of phenolics, although involved in the interaction with the PI3K/Akt/mTOR pathway, proved to be highly engaged in their cytotoxic and antiproliferative activities [54,55].

## 3. Materials and Methods

### 3.1. Sample Collection and Extraction

*P. myrica*, *T. triquetra*, and *S. trinodis* were collected from the intertidal zone in front of the National Institute of Oceanography and Fisheries, Hurghada Red Sea, Egypt, during Autumn 2021. Voucher specimens were kept at the National Institute of Oceanography and Fisheries Herbarium, with codes 20, 30, and 35 for *T. triquetra*, *S. trinodis*, and *P. myrica*, respectively. The three algae were dried, cut into small pieces, and macerated in methanol at room temperature for three days. The extracts were filtered, and the methanol was evaporated under reduced pressure using rotavapor at 45 °C, yielding brown solid residues.

### 3.2. Chemicals and Reagents

Analytical grade methanol for extraction (Nasr Pharmaceutical Company, Cairo, Egypt). LC-MS grade acetonitrile (ACN) and HPLC grade deionized water (DW) were purchased from Merck, Darmstadt, Germany. For the biological study, Dulbecco’s modified Eagle’s was supplemented with 10% heat-inactivated fetal bovine serum (FBS, Thermo Fisher Scientific, GIBCO, Carlsbad, CA, USA; Cat. no. 10099133), 1% penicillin/streptomycin (Thermo Fisher Scientific, Waltham, MA, USA; Cat.no. SV30082), tetrazolium 3-(4,5-dimethylthiazol-2-yl)-2,5-diphenyl-tetrazolium bromide (Gibco-BRL, New York, NY, USA), 2,2-diphenyl-1-picrylhydrazyl (DPPH^•^, Sigma-Aldrich, St. Louis, MO, USA), and 2,2-azino-bis(3-ethylbenzothiazoline-6-sulfonic acid) (ABTS^+^, Sigma-Aldrich, Scotland, UK).

### 3.3. HR LC-MS Analysis and Molecular Networking

The samples were analyzed on an Agilent 1290 Infinity II UPLC coupled to a G6545B Q-TOF MS system with a dual ESI source (Agilent Technologies, Santa Clara, CA, USA). All samples were separated on a Waters ACQUITY UPLC^®^ BEH C18 column (150 × 2.1 mm, 1.7 μm) using 0.1% formic acid–deionized water (A) and ACN (B). The concentration of samples was 1000 ppm. The UPLC solvent gradient systems were optimized as follows: 0–2 min, 5% B; 2–5 min, 5–50% B; 5–20 min, 50–100% B; 20–23 min 100% B; 23–24 min, 100–10% B; 24–25 min, 10% B. The column temperature was set at 30 °C, and the injection volume was 5 μL. The flow rate was 0.3 mL/min. The parameters of the TOF/Q-TOF mass spectrometer were optimized as follows: ion mode, positive- or negative-ion mode; gas temperature, 320 °C; gas flow, 8 L/min; nebulizer pressure, 35 psi; sheath gas temperature, 350 °C; sheath gas flow, 11 L/min; capillary voltage, 3500 V; nozzle voltage, 1000 V; fragmentor voltage, 100 V; MS range, 100–1700 *m*/*z*; MS Acquisition rate, 5 (positive-ion mode) or 3 (negative-ion mode) spectra/s; MS Acquisition time, 200 (positive-ion mode) or 333 (negative-ion mode) ms/spectrum; MS/MS range, 100–1700 (positive-ion mode) 50–1200 (negative-ion mode) *m*/*z*; MS/MS acquisition rate, 5 (positive-ion mode) or 2 (negative-ion mode) spectra/s; MS/MS acquisition time, 200 (positive-ion mode) or 500 (negative-ion mode) ms/spectrum; collision energy fixed, 50 (positive-ion mode) or 15/40 (negative-ion mode); max precursors per cycle, 5. Internal references (purine and HP-0921) were adopted to modify the measured masses in real-time. The reference masses were obtained at *m*/*z* 121.0508/922.0097 and 119.0363/1033.9881 in the positive- and negative-ion mode, respectively. 

A molecular network was created using the online workflow (https://ccms-ucsd.github.io/GNPSDocumentation, accessed on 23 February 2023) on the GNPS website (http://gnps.ucsd.edu, accessed on 23 February 2023). The data were filtered by removing all MS/MS fragment ions within +/−17 Da of the precursor *m*/*z*. MS/MS spectra were window filtered by choosing only the top 6 fragment ions in the +/−50 Da window throughout the spectrum. The precursor ion mass tolerance was set to 0.02 Da and an MS/MS fragment ion tolerance of 0.02 Da. A network was then created where edges were filtered to have a cosine score above 0.5 and more than 3 matched peaks. The edges between 2 nodes were kept in the network if and only if each of the nodes appeared in each other’s respective top 10 most similar nodes. Finally, the maximum size of a molecular family was set to 100, and the lowest-scoring edges were removed from molecular families until the molecular family size was below this threshold. The spectra in the network were then searched against GNPS spectral libraries. The library spectra were filtered in the same manner as the input data. All matches kept between network spectra and library spectra were required to have a score above 0.6 and at least 3 matched peaks.

### 3.4. Assessment of the Antioxidant Activity In Vitro

#### 3.4.1. DPPH Free Radical Scavenging Assay

The free radical scavenging capacity was tested using the DPPH assay, which was modified from the method reported by Burits et al. [56]. Briefly, 1.56–100 µg/mL from each extract was gently mixed with 975 µL of (0.003 g%) DPPH in methanol. After 1 h of dark incubation at room temperature, the absorbance (A) of the reaction mixtures was measured at 515 nm using a Jenway 6305 UV/Vis spectrophotometer (Glasgow, UK). L-Ascorbic acid (20–100 µg/mL) was used as the positive control. Experiments were carried out in triplicates and the DPPH radical scavenging activity (%) was calculated using the following equation:

$$DPPH\;radical\;scavenging\;activity\;\%=\frac{A\;control-A\;sample}{A\;control}\times 100$$

#### 3.4.2. ABTS Radical Scavenging Activity

The ABTS radical scavenging activity was measured following the method described by Re et al. [57]. ABTS radicals were generated by mixing 14 mM ABTS with 4.9 mM potassium persulfate for 16 h in the dark. The ABTS solution was initially diluted with distilled water to reach an absorbance of 0.734 at 734 nm. Then, 975 µL of ABTS^+^ solution was added to 25 µL of algal extract prepared at different concentrations (1.56–100 µg/mL), and the absorbance was measured at 734 nm after 4 min of dark incubation. Experiments were carried out in triplicates and the results were compared with the control, having an absorbance of 0.734 ± 0.12 (ABTS solution only). Ascorbic acid (20–100 µg/mL) was employed as the standard and the ABTS cation radical scavenging activity (%) was estimated using the following equation:

$$ABTS\;radical\;scavenging\;activity\left(\%\right)=\frac{A\;control-A\;sample}{A\;control}\times 100$$

### 3.5. Assessment of the Cytotoxic Activity In Vitro

#### 3.5.1. Cell Line Maintenance and Treatment

The triple-negative breast cancer cell line MDA-231, pancreatic cancer cell line (PANC), estrogen-receptor-positive breast cancer cell line MCF-7, colon cancer cell line (Caco-2), and WISH normal cell line, were seeded with (1 × 10^4^ cells/well) separately using complete media containing Dulbecco’s modified Eagle’s supplemented with 10% heat-inactivated fetal bovine serum, 1% penicillin/streptomycin in a 5% CO_2_ incubator and 95% humidified environment at 37 °C. All cell lines were provided by the Center of Excellence for Research in Regenerative Medicine and Its Applications, Alexandria University, Egypt. The cell lines were incubated with each algal extract at different concentrations (0–200 µg/mL) and with tamoxifen (TAM) as a standard chemotherapeutic drug (0–100 µg/mL) for 48 h. The viability of cells was determined using the tetrazolium 3-(4,5-dimethylthiazol-2-yl)-2,5-diphenyl-tetrazolium bromide MTT assay [58].

#### 3.5.2. Cell Morphology Alterations

Briefly as described by Noser et al. [59], 1 × 10^5^ of the MCF-7 cell line was seeded in a 6-well plate, incubated for 24 h then treated with IC_50_ of the three algal extracts. After 48 h of incubation, morphological alterations of the treated and untreated cells were evaluated and captured using an inverted light microscope (Olympus, Santa Clara, CA, USA).

#### 3.5.3. Cell Cycle Analysis

Flow cytometry was used to analyze cell cycle phases using an Accuri C6 flow cytometer (Becton Dickinson BD, USA) on MCF-7 cells (1 × 10^5^) that were trypsinized, centrifuged at 5000 rpm, then washed with 1× cold phosphate buffer saline (PBS), and fixed with cold absolute ethanol as described by Noser et al. [59] and Darzynkiewicz et al. [60].

#### 3.5.4. Quantitative Real-Time PCR (qRT-PCR)

The MCF-7 (1 × 10^5^) control and treated cells were trypsinized, centrifuged at 4500 rpm, and washed with 1x PBS. The pelleted cells were subjected to RNA extraction and transcription to cDNA as described by Kvastad et al. [61]. On the treated and control cells, the expression of p53 and PI3K mRNA was quantified using Applied qPCR Biosystems (Foster City, CA, USA) according to Livak et al. [62]. Primer 3 plus was used to design the primer sequences, as shown in Table 3.

#### 3.5.5. Western Blot Assessment

For immunoblotting, Mruk and Cheng’s approach [63] was utilized. By cold RIPA lysis buffer, proteins were isolated from the MCF-7 control and treated cells, then quantified using the method established by Bradford [64]. A polyvinylidene difluoride (PVDF) membrane was used to separate equal amounts of proteins (20 µg). After blocking the membrane, phospho-Akt (ab81283) and phospho-mTOR (ab1093) primary antibodies were added and interacted with it. The primary antibodies were then washed several times before treatment with the secondary antibody horseradish peroxidase (HRP) (ab205718). An enhanced chemiluminescence (ECL) detection kit was used to see the bands (Promega, Madison, WI, USA). A gel documentation system (Geldoc-it, UVP, England), was applied for data analysis using Totallab analysis software, ww.totallab.com, Ver.1.0.1 (accessed on 1 March 2021).

#### 3.5.6. Statistical Analysis

The experimental results are presented as mean ± SE. GraphPad Prism 6 software (Boston, MO, USA) was used to determine the significance of differences between the control and treated groups using one-way ANOVA.

## 4. Conclusions

Brown algae are widely disseminated along the Red Sea. LC-MS-guided metabolic studies, assisted by molecular networking, of three Red-Sea-derived brown algae showed their richness in lipids including glycerolipids, fatty acids, and sterol lipids. Despite their relatively low antioxidant power, (especially *S. trinodis* and *T. triquetra*), the algae extracts displayed high and selective antiproliferative activity against MCF-7 cancer cells without showing toxic effects on normal WISH cells. Mechanistic investigations revealed that the algae extracts reduced PI3K expression and activation, hence reducing Akt levels and subsequently increasing p53 levels. The prominent antiproliferative activities of the studied algal extracts are most likely due to the synergistic effect of their chemical metabolites, especially those belonging to classes of fatty acids, glycerolipids, and sterols, constituting the major proportion of the algae extracts with reported cytotoxic and antiproliferative potentials.

## Figures and Tables

**Table 1:** Annotated metabolites in the methanol extracts of the three Red Sea brown algae; namely, *Sirophysalis* (*Cystoseira*) *trinodis*, *Turbinaria triquetra*, and *Polycladia myrica* using LC/ MSD iQ analysis in positive and negative modes.

Peak No.	t_R_ (min)	Annotated Compound	Mono-Isotopic Mass	[M + H]^+^	[M − H]^−^	MS/MS *	Δ ppm	Elemental Composition	Class	Relative Abundance		
										*T.t*	*P.m*	*S.t*
1	1.93	4-Quinolinecarboxylic acid	173.0478	----------	218.0478 [M + HCOO]	200.3843, 166.8669, 154.9239, 122.9169, 106.9795, 93.9133, **79.9573**, 78.9194, 71.0132, 59.0149	0.9	C_10_H_7_NO_2_	Alkaloids	−	−	+
2	2.02	Formononetin	268.0723	----------	267.0655	123, 92, 87, 79, **71**, 59	−0.7	C_16_H_12_O_4_	Phenolics	+	++	−
3	2.08	Galactitol	182.0790	----------	181.0649	135.9147, 96.9595, 79.9576, 71.0141, 60.0176, **59.0145**, 55.0191	0.0	C_6_H_14_O_6_	Sugar alcohol	++	+	+++
4	2.21	Glu-His-Thr	385.1598	----------	384.1456	194.0926, 145.0609, 140.0826, **127.0507**, 109.0404, 82.0299	0.1	C_15_H_23_N_5_O_7_	Peptides/amino acids	+	−	−
5	5.98	N-(3-Oxododecanoyl)-L-homoserine	315.2052	316.2123	----------	128.1084, 123.1159, 115.0380, 103.0934, **102.0913**	2.2	C_16_H_29_NO_5_	Fatty amides	+	++	+
6	6.89	Glycine conjugate sterolipid (ST 19:0;O7;G)	429.2362	430.2428	----------	216.1232, 159.0659, 128.1075, 123.1165, 115.0395, **102.0915**, 100.0480	0.1	C_21_H_35_NO_8_	Sterol lipids	+	+	+
7	7.07	(iso)Loliolide	196.11	197.1173	----------	128.0610, 127.0531, 117.0692, 116.0613, 115.0504, 106.0722, **105.0696**, 103.0536	0.3	C_11_H_16_O_3_	Benzofurans	++	++	+
8	7.07	1-(3,4-Dimethoxyphenyl)-1-propene	178.0994	179.1065	----------	153.0623, 151.0540, 124.9464, 120.0888, 119.0847, 117.0687, **115.0539**, 110.9765, 109.0616, 107.0482, 105.0749, 103.0125	0.1	C_11_H_14_O_2_	Phenolics	+++	++	+
9	8.05	3-Methylsuberic acid (azelaic acid)	188.1049	----------	187.0922	147.5208, 142.9492, 116.0471, 100.9305, 99.9236, 96.2088, 94.6960, 90.0803, 79.9556, **57.0348**	0.2	C_9_H_16_O_4_	Fatty acids	+	−	+
10	8.37	Trimethylphenyl-but-en-one	188.1202	189.1271	----------	141.0692, 129.0702, 128.0608, 127.0525, 116.0585, **115.0533**, 103.0530	0.4	C_13_H_16_O	Phenolics	+	++	−
11	8.61	9,12,13,TriHODE	328.2250	----------	327.2084	211.1330, 171.1024, 97.0659, 85.0295, 71.0140, 69.0350, **57.0348**, 55.0189	0.09	C_18_H_32_O_5_	Fatty acids	+++	+	++
12	8.96	Undecanedioic acid	216.1362	----------	215.1232	164.8686, **142.9528**, 127.8703, 112.9036, 103.9210, 78.9175, 59.9950, 55.0185	0.1	C_11_H_20_O_4_	Fatty acids	+	+	+
13	9.00	Hydroxy-PGE_1_/hydroxy (or keto) PGF_2α_	370.2359	----------	369.2203	139.1127, 121.1034, 115.0411, 109.0654, 99.0453, 95.0510, 83.0512, 69.0350, **59.0145**, 57.0351	0.9	C_20_H_34_O_6_	Prostaglandins	+++	++	+
14	9.09	11,12,13-TriHOME	330.2406	----------	329.2259	211.1341, 183.1399, 171.1030, **139.1130**, 127.1133, 99.0823, 57.0352	−0.06	C_18_H_34_O_5_	Fatty acids	++	+	++
15	9.09	Undecatrienoic acid	180.1153	181.1217	----------	115.0555, 107.0867, 105.0702, 104.0614, **103.0556**	1.4	C_11_H_16_O_2_	Fatty acids	+	++	++
16	9.13	Glucaramide	208.0703	----------	207.0630	**96.9608**, 79.9581	3.6	C_6_H_12_N_2_O_6_	Sugar amide	−	+	−
17	9.30	Prostaglandin E_2_	352.2244	----------	351.2122	167.1065, 147.0818, 125.8720, 85.0297, 83.0512, 69.0349, **59.0147**, 57.0348, 55.0201	−1.6	C_20_H_32_O_5_	Prostaglandins	+	++	+++
18	9.55	Dodecanedioic acid	230.1518	----------	229.1371	166.8675, 138.9444, 109.0867, 99.9254, **79.9582**, 71.0151, 59.0145	−0.04	C_12_H_22_O_4_	Fatty acids	−	+	+
19	10.0	Prostaglandin F_2_	354.2399	----------	353.2246	199.0063, 157.0866, 127.0754, 111.0804, 97.0291, 85.0296, 71.0136, 69.0347, **59.0143**, 57.0350, 55.0202	−2.0	C_20_H_34_O_5_	Prostaglandins	+	++	+++
20	10.07	Carbamoylaminodeoxy hexitol	224.1015	----------	223.0944	**96.9600**, 95.9522, 79.9575	2.9	C_7_H_16_N_2_O_6_	Sugar amide (Urea derivative)	+	+	+
21	10.23	Monoacyl glycerol 20:6	375.2521	376.2594	----------	275.1773, 260.1482, 245.1257, 219.1122, 209.1305, **125.0708**, 118.0770, 105.0702	3.8	C_23_H_35_O_4_	Glycerolipids	−	+	+
22	10.54	Hydroxy-dioxo-heptadecenoic acid	312.1934	----------	311.1785	294.1780, 293.1743, 268.1988, **267.1957**, 249.1849	−0.8	C_17_H_28_O_5_	Fatty acids	−	+	+
23	11.03	Dihydroxyoctadeca trienoic acid	310.2144	----------	309.2065	289.0528, 265.0388, 239.1797, 206.8232, 188.2704, 133.1011, **125.0129**, 114.9469, 110.0361, 107.0501, 102.9777, 99.9258, 78.9592, 55.0192	−0.03	C_18_H_30_O_4_	Fatty acids	+	−	−
24	11.74	Dihydroxy eicosatetraenoic acid	336.2301	----------	335.2147	210.0299, 141.0932, 113.0975, 111.0820, 83.0505, 81.0339, 69.0353, 65.0402, **59.0148**, 57.0346	0.1	C_20_H_32_O_4_	Fatty acids	+	++	+
25	11.79	Diacylglyceryl glucuronides (DGGA 21:4;O)	598.3014	----------	597.2857	297.2419, **225.0059**, 164.9862, 148.9902, 80.9651	4.1	C_30_H_46_O_12_	Glycerolipids	+	−	++
26	11.81	Acrifoline	261.1725	----------	260.1589	147.8940, **118.0681**, 117.0575, 116.0514, 112.9070, 99.9620	−1.4	C_16_H_23_NO_2_	Alkaloids	+	−	−
27	11.81	N-Stearoyl phenylalanine	431.3405	454.3296 [M + Na]^+^	----------	184.0708, 166.0625, 124.9991, 105.1106, **104.1064**	1.3	C_27_H_45_NO_3_	Fatty amides	+	+	+
28	12.23	Antirhine	296.1898	----------	295.1825	183.0159, 136.8927, **134.8963**, 111.0457, 96.9611	3.1	C_19_H_24_N_2_O	Alkaloids	−	+	−
29	12.42	Digalactosyl monoacylglycerols (DGMG 18:3)	676.3670	----------	721.3588 [M + HCOO]^−^	397.1317, 278.2200, **277.2158**, 101.0239, 89.0245, 71.0141, 59.0143	−0.01	C_33_H_56_O_14_	Glycerolipids	+	−	+
30	12.60	Abscisic acid	264.1362	----------	263.1232	98.9566, **96.9604**, 95.9525, 79.9578	0.1	C_15_H_20_O_4_	Isoprenoids	−	−	+
31	12.72	Hydroxyoctadecatetra enoic acid	292.2035	----------	291.1901	205.1147, 117.0854, 164.0810, 132.8977, 96.9607, 79.9561, 71.0500, 68.9946, **59.0140**	−1.1	C_18_H_28_O_3_	Fatty acids	+	−	+
32	13.18	Monoacylglyceryl trimethylhomoserines 20:4	521.3718	522.3787	----------	397.1692, 290.1590, 270.3150, 236.1488, 144.1020, 133.1004, 129.0686, 119.0869, 107.0857, 105.0696, **100.1119**	0.3	C_30_H_51_NO_6_	Glycerolipids	++	+	−
33	13.21	N-acyl glycine 28:6	499.3278	----------	498.3098	438.2954, 349.2120, 168.0418, **78.9590**	−3.9	C_30_H_45_NO_5_	Fatty amides	−	−	+
34	13.52	Hydroxyoctadecatrienoic acid	294.2195	----------	293.2047	221.1526, 140.9423, 134.8966, 127.0773, 113.0600, **96.9604**, 71.0136, 58.0064	0.03	C_18_H_30_O_3_	Fatty acids	++	+	++
35	13.66	Sphinga-4*E*,8*E*,10*E*-trienine	295.2513	296.2586	----------	186.8911, 174.0925, 160.9156, 159.0076, 145.1011, 140.9143, 139.0080, 123.8901, 121.1045, 115.0530, 112.9219, 109.9889, **105.0677**, 103.0524	0.5	C_18_H_33_NO_2_	Sphingolipids	−	+	−
36	13.84	Lysophosphatidylcholines 16:0	495.3325	496.3392	540.3209 [M + HCOO]	227.7908, 197.0506, 184.0729, 166.0620, 145.0023, 124.9995, **104.1070**	0.01	C_24_H_50_NO_7_P	Phospholipids	++	−	+
37	14.06	Sulfoquinovosyl monoacylglycerols (SQMG 14:0)	528.2598	----------	527.2430	225.0076, **80.9658**	−1.1	C_23_H_44_O_11_S	Glycerolipids	++	+	++
38	14.12	Hydroxyeicosapentaenoic acid	318.2195	----------	317.2043	173.1326, 121.0665, 91.0544, 71.0158, **59.0152**	0.03	C_20_H_30_O_3_	Fatty acids	++	+	++
39	14.17	Dihydroxyhexadecanoic acid	288.2293	----------	287.2152	211.2046, 183.1742, 181.1582, 85.0284, 84.0208, 71.0502, **57.0344**, 55.0190	−2.6	C_16_H_32_O_4_	Fatty acids	+	+++	++
40	14.32	Sulfoquinovosyl monoacylglycerols (SQMG 18:3)	578.2760	----------	577.2617	**225.0060**, 80.9649	−0.1	C_27_H_46_O_11_S	Glycerolipids	++	+	++
41	14.37	Monoacylglyceryl trimethylhomoserines 16:0	473.3719	474.3785	----------	236.1487, 144.1023, **100.1119**	0.4	C_26_H_51_NO_6_	Glycerolipids	++	+++	+
42	14.38	Eicosapentaenoic acid	302.2244	303.2314	301.2101	131.0843, 129.0694, 117.0699, 115.0540, **105.0692**, 103.0540	0.5	C_20_H_30_O_2_	Fatty acids	++	+	++
43	14.39	Monogalactosylmonoacylglycerols (MGMerolG 18:3)	514.3143	----------	549.2762 [M + Cl]^−^	301.2133, 249.0603, 113.0236, 99.0083, 85.0297, 75.0086, **59.0139**	0.2	C_27_H_46_O_9_	Glycerolipids	++	−	+
44	14.47	Ether lysophosphatidyl choline (LPC O-16:0)	481.3533	482.3602	----------	184.0719, 166.0609, 124.9993, **104.1065**	2.0	C_24_H_52_NO_6_P	Phospholipids	+	−	++
45	14.51	Sterol–sulfate conjugates (ST 28:2;O3;S)	510.3040	----------	509.2859	452.3132, 363.2293, 228.2060, **227.2018**, 78.9593, 73.0309, 59.0143	4.8	C_28_H_46_O_6_S	Sterol lipids	+	−	−
46	14.56	Monogalactosylmonoacylglycerols (MGMG 14:0)	464.2983	----------	499.2571 [M + Cl]^−^	452.3152, 363.2314, 174.9689, 168.0443, **78.9598**	−0.4	C_23_H_44_O_9_	Glycerolipids	+	−	−
47	14.65	Sphingadienine	297.2667	298.2738	----------	235.0083, 193.1011, 171.0918, 154.0726, 133.1020, 128.0639, 119.0881, 109.0641, **105.0699**, 100.9376	−0.2	C_18_H_35_NO_2_	Sphingolipids	+	+++	++
48	14.83	Monoacylglyceryl trimethylhomoserines 18:1	499.3874	500.3947	----------	236.1487, 144.1017, 109.1024, **100.1113**	0.2	C_28_H_53_NO_6_	Glycerolipids	++	+	−
49	15.04	Sulfoquinovosyl monoacylglycerols (SQMG 27:5)	700.3874	----------	699.3702	397.1343, 256.2359, **255.2323**, 101.0245, 89.0245, 71.0141, 59.0145	2.5	C_36_H_60_O_11_S	Glycerolipids	++	+	++
50	15.06	Digalactosylmonoacylglycerols (DGMG 16:0)	654.3822	----------	689.3412 [M + Cl]^−^	397.1337, 256.2352, **255.2324**, 113.0244, 101.0246, 89.0243, 59.0143	−0.7	C_31_H_58_O_14_	Glycerolipids	+	+	+
51	15.11	N-Palmitoyl tryptophan	442.3182	----------	477.2764 [M + Cl]^−^	321.2049, **255.2308**, 160.8407, 78.9589, 73.0297, 59.0139, 57.0351, 55.0195	−3.0	C_27_H_42_N_2_O_3_	Fatty amides	++	+	++
52	15.21	N-(Tridecanoyl)-4*E*-tetradecasphingenine (C_14_ Ceramide)	439.4027	440.4093	----------	284.2660, 283.2626, 103.0938, **102.0910**	0.3	C_27_H_53_NO_3_	Sphingolipids	++	++	+
53	15.34	Deoxysphingatetraenine	277.2405	278.2477	----------	139.0064, 129.0681, 128.0604, 117.0682, 107.0841, **105.0692**	−0.2	C_18_H_31_NO	Sphingolipids	+++	+	++
54	15.39	Diacylglyceryl glucuronides 16:0	660.445	683.4334	----------	598.3259, **597.3243**, 435.2182, 419.2218, 287.1465, 266.9982, 243.1199, 207.0322, 155.0679, 147.0655, 126.0652	0.2	C_35_H_64_O_11_	Glycerolipids	−	+	−
55	15.45	Monogalactosyldiacylglycerols 24:1	616.419	639.4068 [M + Na]^+^	----------	554.3029, **553.2985**, 375.1994, 347.1693, 243.1198, 215.0902, 200.1027, 199.0941, 156.0745, 155.0663, 111.0419	0.5	C_33_H_60_O_10_	Glycerolipids	+	+	+
56	15.58	Sterol–hexose conjugates (ST 20:0;O2;Hex)	468.3084	----------	503.2746	254.2220, **253.2176**, 85.0301, 75.0097, 59.0152	−0.6	C_26_H_44_O_7_	Sterol lipids	−	+	−
57	15.58	Hydroxyeicosatetraenoic acid	320.2351	----------	319.2211	301.2177, 275.2381, 168.1118, **167.1085**, 149.0978, 59.0148	−0.1	C_20_H_32_O_3_	Fatty acids	+++	+	++
58	15.75	Arg-Lys-Lys tripeptide	430.3037	----------	429.2899	117.0661, 99.0558, **87.0558**, 74.0243, 58.0302	4.8	C_18_H_38_N_8_O_4_	Peptides/amino acids	−	+	−
59	15.77	Sterol 27:0;O7	484.3401	507.3291 [M + Na]^+^	----------	**421.2207**, 381.1921, 259.1161, 243.1188, 171.0624, 155.0675, 142.0599, 127.0367, 112.0499, 111.0412	0.1	C_27_H_48_O_7_	Sterol lipids	+	+++	++
60	15.84	Sterol 25:0;O6	440.3138	463.3029 [M + Na]^+^	----------	377.1955, 337.1600, 215.0877, 199.0948, **127.0357**, 111.0419	0.02	C_25_H_44_O_6_	Sterol lipids	++	+	+
61	15.98	Monoacylglyceryl glucuronides (MGGA 20:4)	554.3080	----------	553.2888	303.2331, 281.2492, 113.0248, 99.0093, 85.0298, 75.0094, 71.0143, **59.0148**	−1.9	C_29_H_46_O_10_	Glycerolipids	−	+	−
62	16.06	Monogalactosylmonoacylglycerols (MGMG 18:2)	516.3298	----------	561.3186 [M + HCOO]	280.2359, **279.2327**, 59.0145	−0.05	C_27_H_48_O_9_	Glycerolipids	++	+	+
63	16.07	Hydroxyoctadecadienoic acid	296.2351	----------	295.2200	280.2346, **279.2315**, 253.0910, 101.0241, 71.0139, 59.0145	−0.1	C_18_H_32_O_3_	Fatty acids	+	++	+++
64	16.08	Phosphatidylglycerol (PG 20:1)	552.3057	----------	551.2901	303.2323, 113.0243, 99.0089, 85.0297, 75.0093, 71.0143, **59.0144**	−1.1	C_26_H_49_O_10_P	Phospholipids	+	−	−
65	16.18	Dictyone acetate	286.23	287.2361	----------	143.0854, 142.0777, 131.0845, 130.0758, 119.0850, 117.0692, 115.0533, **105.0696**	1.15	C_20_H_30_O	Diterpenes	−	+	−
66	16.49	Hydroxyoctadecenoic acid	298.2507	----------	297.2368	184.0132, **183.0110**, 134.8942, 119.0501	−0.3	C_18_H_34_O_3_	Fatty acids	+	+	++
67	16.57	Monogalactosylmonoacylglycerols (MGMG 16:1)	490.3133	----------	489.2947	280.2357, **279.2326**, 78.9595	−1.7	C_25_H_46_O_9_	Glycerolipids	−	−	++
68	16.57	Acyl carnitines (CAR 11:0)	329.2563	----------	328.2410	185.0158, **74.0248**, 72.0451, 59.0142, 56.0158	−0.9	C_18_H_35_NO_4_	Fatty amides	−	+	−
69	16.58	Citric acid stearyl ester	444.3077	----------	479.2708 [M + Cl]^−^	323.2554, **305.2472**, 279.2300, 253.2164, 223.1679, 162.8376, 78.9595, 73.0291, 57.0343, 55.0196	−2.2	C_24_H_44_O_7_	Fatty esters	+	−	++
70	16.86	Hydroxynonacosa heptaenoic acid	440.3297	441.3349	----------	185.1320, 159.1153, 157.1016, 145.1003, 143.0844, 137.0952, 131.0851, 129.0698, 119.0852, 107.0862, 106.0721, **105.0689**	1.4	C_29_H_44_O_3_	Fatty acids	+	−	−
71	16.91	Sulfoquinovosyl monoacylglycerols (SQMG 15:0)	542.2748	----------	541.2592	225.0065, 164.9856, 94.9810, **80.9653**	−2.3	C_24_H_46_O_11_S	Glycerolipids	+	−	+
72	16.99	4-Dodecylphenol	262.2306	263.2370	----------	160.0537, 145.0977, 122.0892, 118.0739, 116.0610, 115.0537, 108.9955, **105.0694**, 104.0583, 103.0550	−3.4	C_18_H_30_O	Phenolics	+	++	++
73	17.11	Linoleamide	279.2569	280.2636	----------	133.1010, 119.0852, 109.1007, 107.0849, **105.0693**, 103.0534	2.4	C_18_H_33_NO	Fatty amides	++	+++	+
74	17.13	Hydroxyeicosadienoic acid (FA 20:2;OH)	324.2657	----------	323.2511	223.1702, 221.1930, 199.1308, **183.0101**, 181.1193, 71.0143, 57.0349	−2.3	C_20_H_36_O_3_	Fatty acids	++	+	+
75	17.24	N-(Pentadecanoyl) tetradecasphingenine (Ceramide d18:0/15:0)	467.4339	468.4411	----------	312.2969, 311.2938, 175.0714, 128.1070, 123.1173, 109.1005, 103.0940, **102.0912**	0.1	C_29_H_57_NO_3_	Sphingolipids	++	++	+
76	17.38	Sulfoquinovosyl monoacylglycerols (SQMG 18:2)	580.2911	----------	579.2757	**225.0066**, 80.9655	−1.0	C_27_H_48_O_11_S	Glycerolipids	++	+	++
77	17.70	Palmitoyl-galactosylglycerol	492.3289	----------	527.2907 [M + Cl]^−^	281.2491, 256.2376, **255.2327**, 113.0257, 95.0138, 89.0238, 85.0284, 83.0135, 71.0137, 59.0141	−1.8	C_25_H_48_O_9_	Glycerolipids	++	+	++
78	17.72	Lauroylcarnitine	343.2714	----------	342.2570	75.0289, **74.0252**, 72.0447	−2.5	C_19_H_37_NO_4_	Fatty amides	−	+	−
79	17.81	Sterol–glycine conjugate (22:5;O3;G)	397.2254	398.2326	----------	266.0807, 252.0648, 240.1019, 224.0705, 164.0699, 150.0259, **149.0229**, 121.0278	0.2	C_24_H_31_NO_4_	Sterol lipids	−	+	−
80	17.83	Monoacylglyceryl glucuronides (MGGA 16:0)	506.3077	----------	505.2927	256.2346, **255.2314**, 85.0294, 75.0090, 71.0141, 59.0141	−2.7	C_25_H_46_O_10_	Glycerolipids	++	+	++
81	18.05	N-Acyl ornithines (20:1)	454.3389	----------	453.3264	255.2287, **145.0603**, 127.0499, 125.0702, 109.0395, 101.0707, 84.0455, 59.0299	−3.8	C_25_H_46_N_2_O_5_	Fatty amides	−	+	−
82	18.08	Dihydroxyoctadecenoic acid	314.2446	----------	313.2321	72.9923, 58.0034, **56.9981**	−3.5	C_18_H_34_O_4_	Fatty acids	−	+	-
83	18.17	Palmitoyl hexitol	420.3080	----------	419.2913	256.2357, **255.2321**, 59.0144, 55.0199	−1.6	C_22_H_44_O_7_	Fatty esters	+	−	+
84	18.19	Fatty acid ester of hydroxy fatty acids (30:8;O)	466.3066	----------	465.2987	256.2357, **255.2325**, 101.0243, 59.0148	−3.6	C_30_H_42_O_4_	Fatty esters	+	+	++
85	18.21	Sterol (27:3;O6)	462.2981	463.3028	----------	260.2020, 243.2076, 219.1703, 199.1495, 171.1156, 157.1009, 145.1008, 143.0851, 133.1001, 131.0853, 129.0683, 119.0847, 117.0696, 109.1026, 107.0845, 106.0720, **105.0696**	0.11	C_27_H_42_O_6_	Sterol lipids	+	+	−
86	18.40	Monoacylglyceryl glucuronides (MGGA 18:1)	532.3244	----------	531.3102	282.2497, **281.2466**, 85.0295, 75.0087, 71.0140, 59.0142	−0.6	C_27_H_48_O_10_	Glycerolipids	+	++	+
87	18.42	Sterol–hexose conjugates (ST 21:3;O;Hex)/Sterols (ST 27:4;O6)	460.2823	----------	459.2677	416.3226, 415.3227, 387.3238, 157.0501, 127.8685, 73.0308, 59.0147, **55.0204**	−0.4	C_27_H_40_O_6_	Sterol lipids	+	+	++
88	18.42	Hexose ceramide 37:4;O4	767.5544	768.5610	----------	**750.5538**, 751.5564, 512.3231, 474.3797, 456.3696, 277.1792, 144.1024, 100.1127	0.4	C_43_H_77_NO_10_	Sphingolipids	−	+	−
89	18.85	Sterol–hexose conjugates (ST 19:1;O;Hex)/Sterols (ST 25:2;O6)	436.2816	----------	435.2679	277.2145, 157.0506, 101.0226, 85.0287, 73.0290, 59.0139, 57.0346, **55.0191**	−2.0	C_25_H_40_O_6_	Sterol lipids	+	+	++
90	19.00	Sterols (ST 29:3;O5)	474.3322	----------	473.3190	430.3405, **429.3366**, 411.3264, 371.2575	−4.8	C_29_H_46_O_5_	Sterol lipids	++	−	+
91	19.04	Palmitoylalanine	327.2768	----------	326.2624	221.1506, 211.1328, 184.0131, 139.9472, 117.0757, **116.0720**, 98.9624, 59.0140	−1.6	C_19_H_37_NO_3_	Fatty amides	+	−	+
92	19.17	Oleamide	281.2733	282.2795	----------	125.0946, 121.0998, 119.0857, 111.0808, 109.1008, 108.0858, **107.0854**, 106.0738, 105.0704, 100.0754	5.0	C_18_H_35_NO	Fatty amides	++	+	+
93	19.41	Deoxyerythronolide B	386.2660	----------	385.2516	227.2004, **116.0720**, 68.9967, 57.0352, 55.0197	−2.1	C_21_H_38_O_6_	Macrolides (polyketides)	+	+	+
94	19.50	Octadecatrienoic acid	278.2237	----------	277.2104	131.7441, 96.9583, 96.0531, 92.9945, **71.0140**, 58.0062	−3.1	C_18_H_30_O_2_	Fatty acids	++	−	+
95	19.52	Oleoyl alanine/Palmitoyl proline	353.2918	----------	352.2787	126.0928, 117.0754, **116.0715**, 68.0507	−3.3	C_21_H_39_NO_3_	Fatty amides	++	+	+++
96	19.62	Sterols (ST 23:0;O6)	412.2816	----------	411.2663	254.2205, **253.2173**, 101.0241, 73.0302, 57.0354, 55.0197	−2.1	C_23_H_40_O_6_	Sterol lipids	+	+	++
97	19.65	Eicosatetraenoic acid	304.2400	----------	303.2259	236.7105, 159.2504, 127.0726, 121.0501, 115.4655, 108.3766, 96.9600, **68.9949**, 62.5142, 59.0137	−0.7	C_20_H_32_O_2_	Fatty acids	+	+	++
98	19.74	Sterol–hexose conjugate (ST 21:2;O;Hex)/sterols (ST 27:3;O6)	462.2974	----------	461.2827	304.2349, **303.2319**, 175.0609, 157.0503, 73.0300, 55.0197	−1.6	C_27_H_42_O_6_	Sterol lipids	+++	+	++
99	20.11	Phaeophorbide a	592.2686	593.2758	----------	534.2569, 533.2543, 505.2226, 462.2338, 461.2309, **460.2219**, 447.2172, 433.2351	0.04	C_35_H_36_N_4_O_5_	Tetrapyrroles	+	−	−
100	20.23	Sterols (ST 28:3;O5)	460.3180	----------	459.3012	398.3094, **397.3075**, 379.2977, 369.3125, 305.2464, 123.0445, 95.0503, 57.0345, 55.0189	−1.8	C_28_H_44_O_5_	Sterol lipids	+	+	+++
101	20.29	Sterol–hexose conjugate (ST 19:0;O;Hex)	438.2971	----------	437.2822	280.21356, **279.2320**, 73.0302, 57.0352, 55.0199	−2.3	C_25_H_42_O_6_	Sterol lipids	+	+	+
102	20.30	N-Oleoyl GABA	367.3076	----------	366.2920	183.0122, **130.0881**	−2.8	C_22_H_41_NO_3_	Fatty amides	++	+	++
103	20.91	N-Undecyl benzenesulfonic acid	312.1748	----------	311.1611	184.0163, **183.0115**, 123.9703	−3.5	C_17_H_28_O_3_S	Benzene sulfonic acids	+	−	−
104	21.01	Monogalactosylmonoacylglycerols 30:4	680.486	703.4755 [M + Na]^+^	----------	615.3830, 527.2961, 463.2650, 375.1778, **335.2164**, 247.1290, 139.0323, 101.0695	−0.4	C_39_H_68_O_9_	Glycerolipids	+	−	−
105	21.02	DGCC (Diacylglyceryl-3-O-carboxyhydroxy methylcholines) 29:2;O	697.5119	698.5194	----------	175.1478, 159.1144, 149.1297, 147.1148, 145.1004, 135.1148, 133.1006, 131.0828, 125.0943, 121.0997, 119.0830, 117.0677, **109.0996**, 107.0838, 105.0684	−1.4	C_39_H_71_NO_9_	Glycerolipids	+	−	−
106	21.09	Hydroxynonacosa hexaenoic acid	442.3438	443.3516	----------	173.1319, 145.0985, 133.0991, 123.0784, 119.0835, 111.0777, 110.0677, **109.0633**, 107.0839, 105.0690	−2.0	C_29_H_46_O_3_	Fatty acids	+	−	+
107	21.32	Octadecanamide	283.2875	567.5816 [2M + H]^+^	----------	285.2970, **284.2927**, 102.0893	0.03	C_18_H_37_NO	Fatty amides	+	++	+
108	21.44	Monoacylglyceryl glucuronides (MGGA 22:3)	584.3541	----------	583.3393	256.2356, **255.2315**, 157.0494, 125.0602, 59.0147	−3.3	C_31_H_52_O_10_	Glycerolipids	+	−	−
109	21.45	Oxidized phospatidylglycerol (PG 22:4;O3)	622.2778	623.2852	----------	574.2479, 546.2553, **545.2532**, 517.2588, 503.2439, 486.2346, 485.2311, 477.2273, 459.2165, 422.2411, 407.2236	3.8	C_28_H_47_O_13_P	Phospholipids	++	+	−
110	21.55	Sulfoquinovosyl monoacylglycerols (SQMG 16:0)	556.2893	----------	555.2737	299.0420, 225.0071, 164.9866, 148.9913, 94.9811, **80.9658**	−4.3	C_25_H_48_O_11_S	Glycerolipids	++	+	++
111	21.62	N-Palmitoyl valine	355.3080	----------	354.2921	308.5868, 159.0427, 126.0897, 117.0752, 116.9299, **116.0729**, 114.0935	−1.8	C_21_H_41_NO_3_	Fatty amides	++	+	++
112	21.98	Sterol (ST 25:0;O6)	440.3120	----------	439.2980	282.2491, **281.2454**, 73.0292, 57.03418, 55.0193	−4.0	C_25_H_44_O_6_	Sterol lipids	++	+	+
113	22.29	N-Palmitoyl(iso)leucine	369.3232	----------	368.3091	324.3302, 254.2527, 239.2385, 131.0898, **130.0878**, 91.5255, 82.0659	−2.9	C_22_H_43_NO_3_	Fatty amides	+	−	+
114	22.50	Hydroxyeicosanoic acid	328.2966	----------	327.2813	185.0134, 139.9439, 68.9964, 61.9895, **59.0150**	−3.5	C_20_H_40_O_3_	Fatty acids	++	+++	−
115	22.68	Sterols (ST 21:6;O5)	356.1641	----------	355.1484	117.9297, **116.9299**, 100.9339, 99.9274, 84.9412	−4.6 or 4.8	C_18_H_28_O_5_S or C_21_H_24_O_5_	Sterol lipids	+	+	−
116	22.77	Octadecenoic acid (Oleic acid)	282.2550	----------	281.2445	**96.9581**, 85.1317, 68.9957, 59.0138	−3.1	C_18_H_34_O_2_	Fatty acids	+	+	−
117	22.80	Sterols (ST 28:4;O5)	458.3014	----------	457.2890	**369.3116**, 367.2897, 353.2803, 325.1785, 139.9720, 110.9835, 95.0505	−3.9	C_28_H_42_O_5_	Sterol lipids	++	+	−
118	22.97	Monoacylglyceryl glucuronides (MGGA 18:5)	524.2620	----------	523.2454	255.2320, **80.9659**, 71.0147	−0.2	C_27_H_40_O_10_	Glycerolipids	−	−	+

* Base peak fragment is assigned in bold. Identification of metabolites was achieved using MassHunter, MassBank, GNPS, LIPIDMAPS, metabolomics workbench, PubChem, ChemSpider, and from the literature data.

**Table 2:** The anti-proliferative activity of the three Red Sea algal extracts *S. trinodis*, *P. myrica*, and *T. triquetra*.

Cell Line	IC_50_ (µg/mL)			
	*S. trinodis*	*P. myrica*	*T. triquetra*	Tamoxifen
MCF-7	51.37 ± 1.19	63.44 ± 1.13	59.70 ± 1.22	38.53 ± 1.11
MDA-231	69.41 ± 1.16	130.40 ± 1.13	67.22 ± 1.26	48.35 ± 1.11
CaCo-2	100.10 ± 1.14	114.60 ± 1.12	90.75 ± 1.22	42.18 ± 1.12
PANC-1	105.80 ± 1.16	157.80 ± 1.28	110 ± 1.22	52.19 ± 1.16
WISH	196.30 ± 1.26	200 ± 1.15	198.60 ± 1.12	30.62 ± 1.16

Experiment was carried out in triplicates and IC_50_ is expressed as µg/mL ± SE.

**Table 3:** Primer sequences used for qRT-PCR.

Gene	Forward Primer (/5–/3)	Reverse Primer (/5–/3)
P53	TAACAGTTCCTGCATGGGCGGC	AGGACAGGCACAAACACGCACC
PI3K	GCTCTCTCACTGCATACATTGT	AGTCACAGCTGTATTGGTCG
GAPDH	TGTGTCCGTCGTGGATCTGA	CCTGCTTCACCACCTTCTTGA

## Data Availability

The data presented in this study are available in the article or Supplementary Material.

## Supplementary Materials

The following supporting information can be downloaded at: https://www.mdpi.com/article/10.3390/md21070404/s1. Figure S1. Anti-p-Akt antibody protein expression level for the algae extracts; Figure S2. Computerized analysis of anti-p-Akt antibody protein expression level for the algae extracts; Figure S3. Dendrograms of anti-p-Akt antibody protein expression level for all samples; Figure S4. Anti-p-mTOR antibody protein expression level for algae extracts; Figure S5. Computerized analysis of anti-p-mTOR antibody protein expression level for algae extracts; Figure S6. Dendrograms of anti-p-mTOR antibody protein expression level for all samples; Figure S6. TIC chromatogram of the extract of the Red Sea algae *T. triquetra* in positive mode; Figure S7. TIC chromatogram of the extract of the Red Sea algae *P. myrica* in positive mode; Figure S8. TIC chromatogram of the extract of the Red Sea algae *S. trinodis* in positive mode; Figure S9. Total ion chromatogram of the extract of the Red Sea algae *S. trinodis* in positive mode; Table S1. The expression level of anti-p-Akt protein expression level for algae extracts; Table S2. The expression level of anti-p-mTOR protein expression level for algae extracts.
