# Supplementary material for: Enhanced Expression of p53 and Suppression of PI3K/Akt/mTOR by Three Red Sea Algal Extracts: Insights on Their Composition by LC-MS-Based Metabolic Profiling and Molecular Networking
Source: Mar Drugs. 2023 Jul 17;21(7):404. doi: 10.3390/md21070404 (PMC10381385; doi:10.3390/md21070404)
Supplement: Supplementary file 1 [file marinedrugs-21-00404-s001.zip › marinedrugs-2501414-supplementary.pdf]

Supplementary file

Enhanced Expression of p53 and Suppression of PI3K/Akt/mTOR by Three Red Sea Algal Extracts: Insights on their Composition by LC-MS-based Metabolic Profiling and Molecular Networking

Nouran M Fahmy [1#], Mariam I Gamal El-Din [1#], Maha M Salem [2], Sarah H Rashedy [3], Gyu Sung Lee [4], Yoon Seo Jang [5], Ki Hyun Kim [5], Chung Sub Kim [4,5,*], Mohamed El-Shazly [1*], Shaimaa Fayez [1]

[1] Department of Pharmacognosy, Faculty of Pharmacy, Ain-Shams University, Cairo 11566, Egypt; nouran_fahmy@pharma.asu.edu.eg, mariam_gamal@pharma.asu.edu.eg, mohamed.elshazly@pharma.asu.edu.eg, shaimaa_fayez@pharma.asu.edu.eg.
[2] Biochemistry Division, Chemistry Department, Faculty of Science, Tanta University, Tanta 31527, Egypt; maha_salem@science.tanta.edu.eg.
[3] National Institute of Oceanography and Fisheries (NIOF), Cairo 11516, Egypt; sarahamdy.niof@gmail.com
[4] Department of Biopharmaceutical Convergence, Sungkyunkwan University, Suwon, 16419, Republic of Korea; dlrbtjd36@skku.edu, chungsub.kim@skku.edu
[5] School of Pharmacy, Sungkyunkwan University, Suwon 16419, Republic of Korea; bbj0423@g.skku.edu, khkim83@skku.edu, chungsub.kim@skku.edu

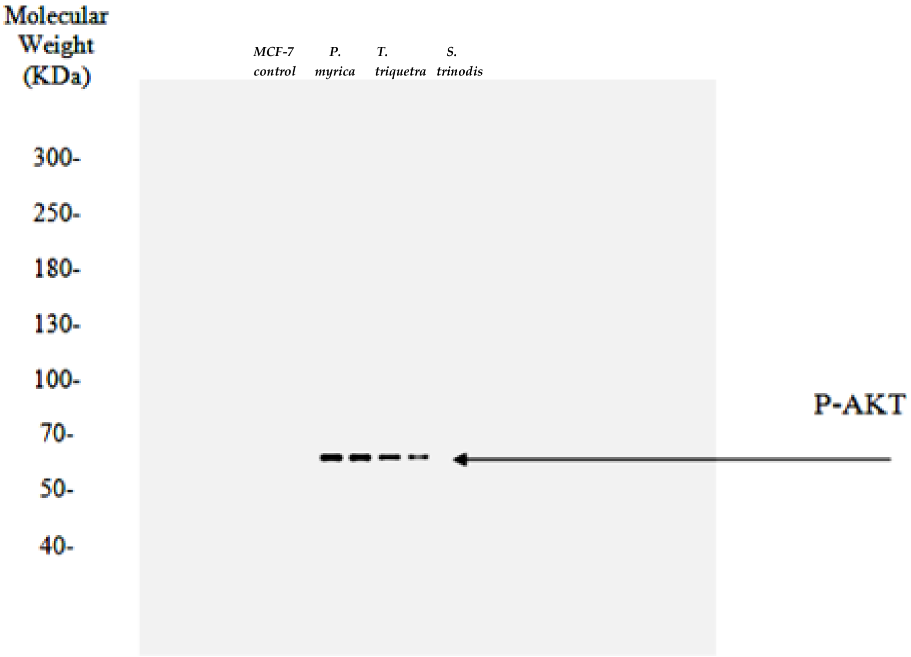

Figure S1. Ant-p-Akt antibody protein expression level for algae extracts.The whole raw data immunoblotting gel before cropping

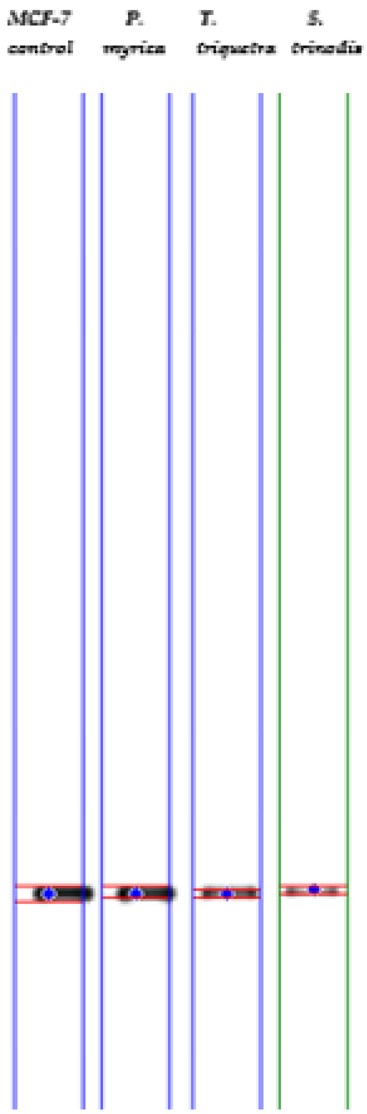

Figure S2. Computerized bands analysis of anti-p-Akt antibody protein expression level for algae extracts.

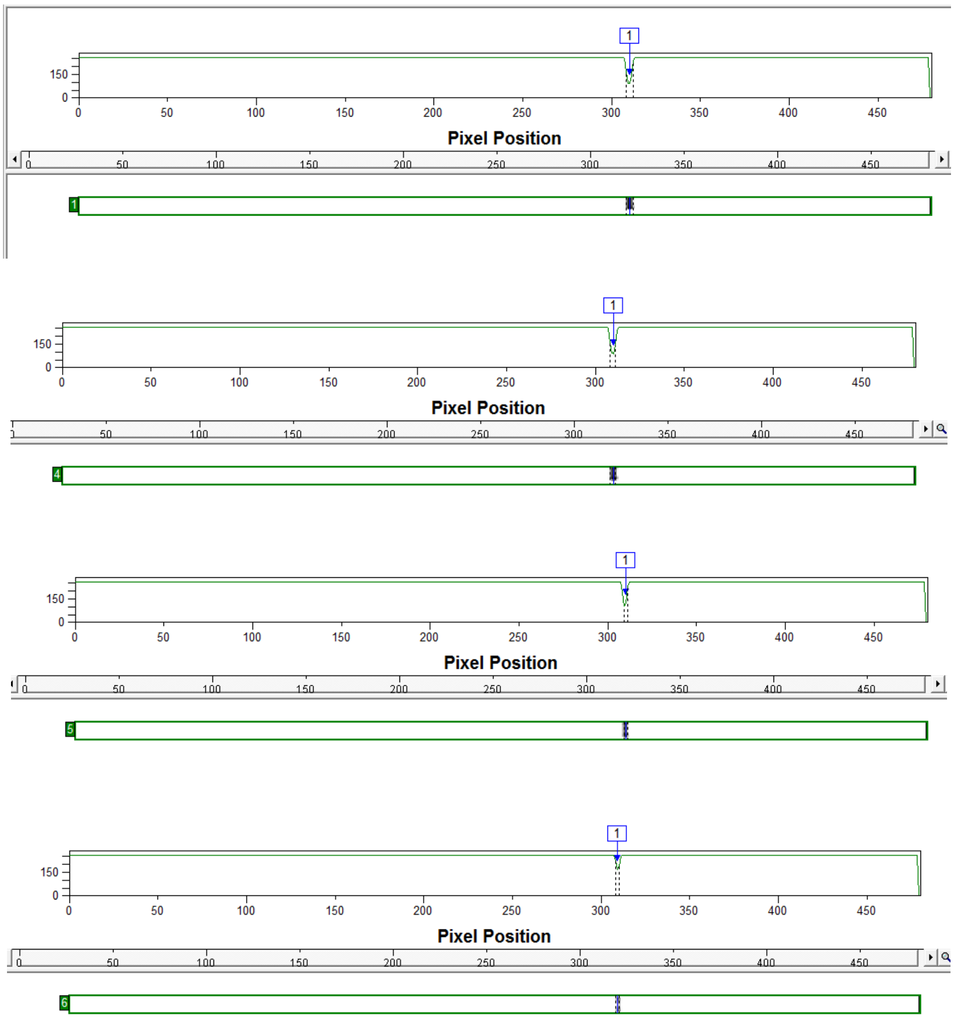

Figure S3. Dendrograms of anti-p-Akt antibody protein expression level bands analysis for all algae extracts.

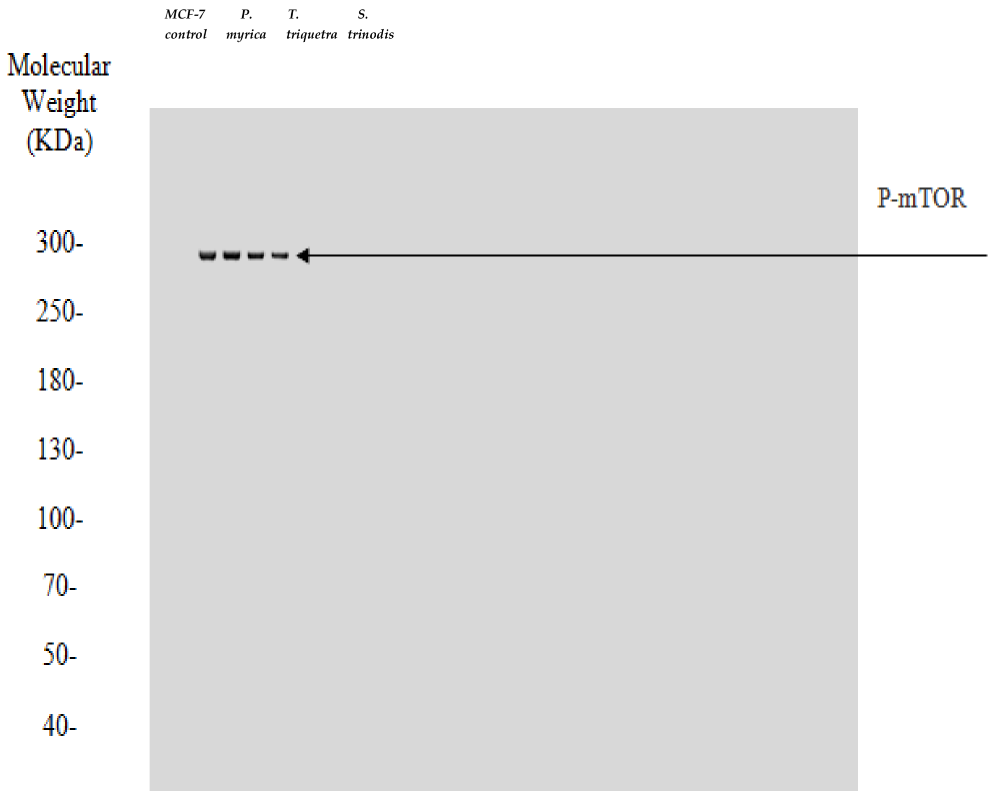

Figure S4. Anti-p-mTOR antibody protein expression level for algae extracts. The whole raw data immunoblotting gel before cropping

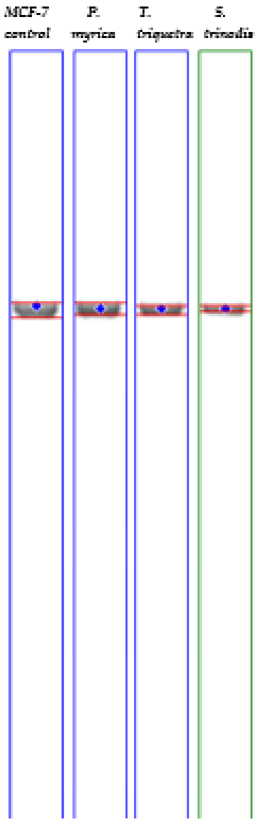

Figure S5. Computerized bands analysis of anti-p-mTOR antibody protein expression level for algae extracts.

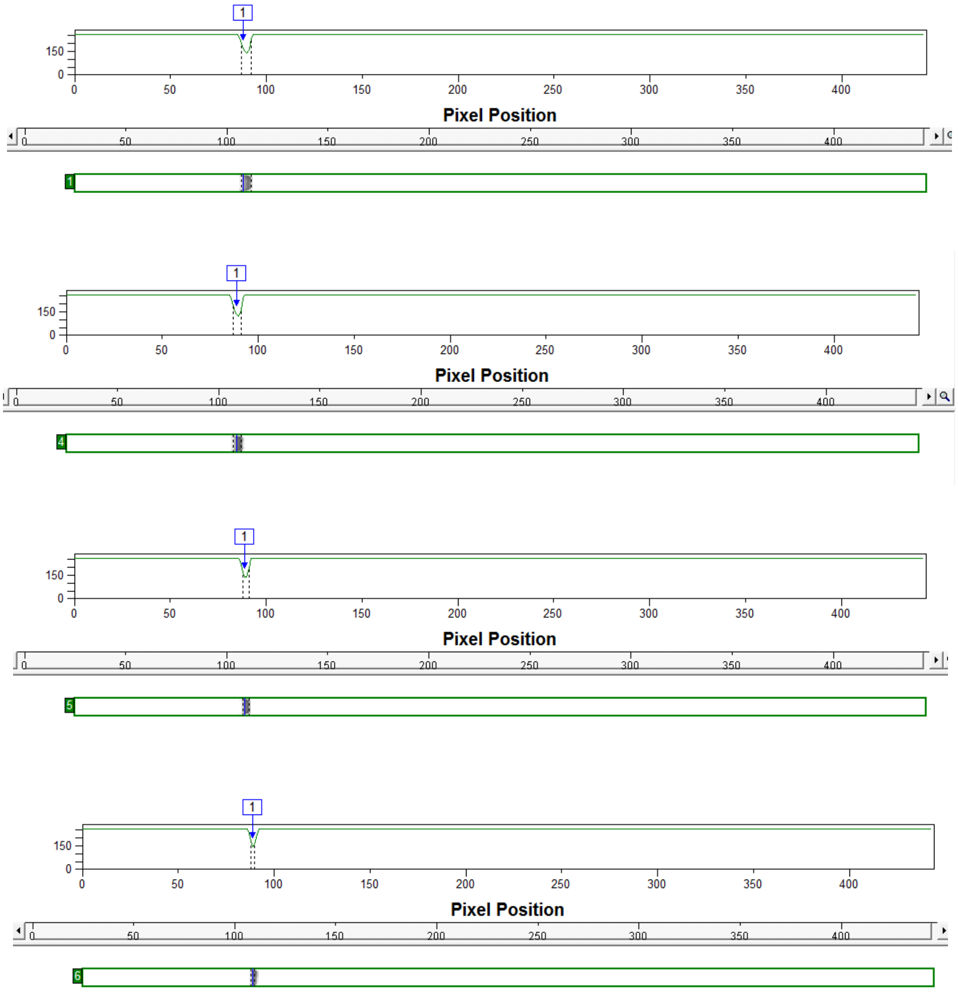

Figure S6. Dendrograms of anti-p-mTOR antibody protein expression level band analysis for algae extracts.

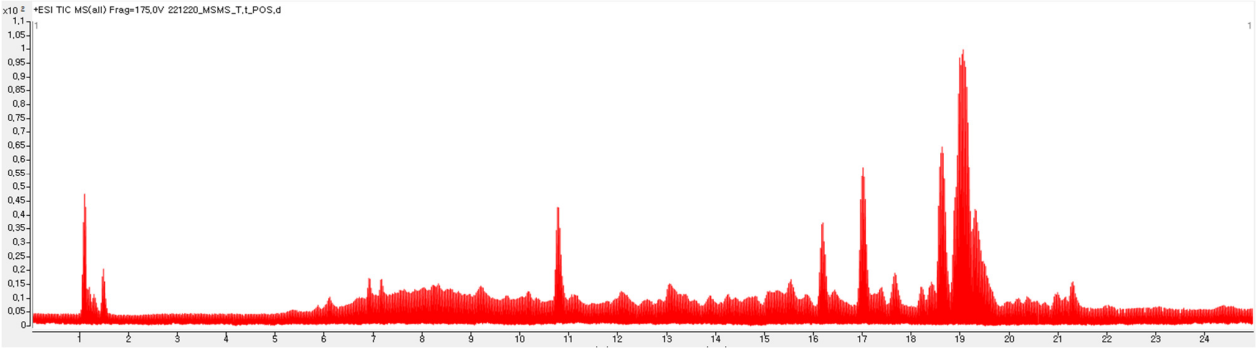

Figure S7. Total ion chromatogram of the extract of the Red Sea algae *T. triquetra* in positive modes.

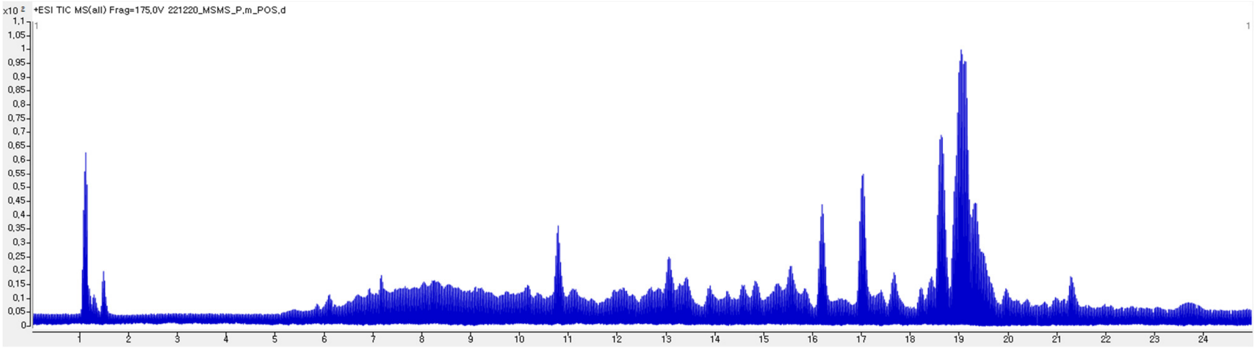

Figure S8. Total ion chromatogram of the extract of the Red Sea algae *P.myrica* in positive mode.

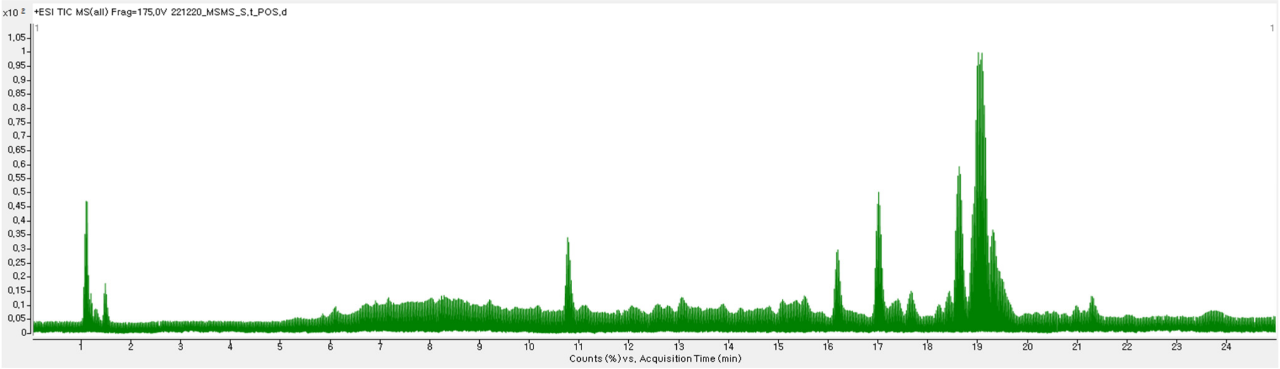

Figure S9. Total ion chromatogram of the extract of the Red Sea algae *S. trinodis* in positive mode.

Table S1. The expression level of anti-p-Akt protein bands after normalized with *β*-actin for algae extracts.

MCF-7 control	*P. myrica*	*T. triquetra*	*S. trinodis*
Lane %	Lane %	Lane %	Lane %
0.80	0.48	0.35	0.29

Table S2. The expression level of anti-p-mTOR protein bands after normalized with *β*-actin for algae extracts.

MCF-7 control	*P. myrica*	*T. triquetra*	*S. trinodis*
Lane %	Lane %	Lane %	Lane %
0.72	0.53	0.39	0.28
